# Comparative transcriptome analysis to identify putative genes involved in carvacrol biosynthesis pathway in two species of *Satureja*, endemic medicinal herbs of Iran

**DOI:** 10.1371/journal.pone.0281351

**Published:** 2023-07-07

**Authors:** Somayeh Shams, Ahmad Ismaili, Farhad Nazarian Firouzabadi, Hasan Mumivand, Karim Sorkheh

**Affiliations:** 1 Faculty of Agriculture, Department of Plant Production and Genetic Engineering, Lorestan University, Khorramabad, Iran; 2 Faculty of Agriculture, Department of Horticultural Science, Lorestan University, Khorramabad, Iran; 3 Faculty of Agriculture, Department of Plant Production and Genetic Engineering, Shahid Chamran University of Ahvaz, Ahvaz, Iran; Waterford Institute of Technology, IRELAND

## Abstract

*Satureja* is rich in phenolic monoterpenoids, mainly carvacrol, that is of interest due to diverse biological activities including antifungal and antibacterial. However, limited information is available regarding the molecular mechanisms underlying carvacrol biosynthesis and its regulation for this wonderful medicinal herb. To identify the putative genes involved in carvacrol and other monoterpene biosynthesis pathway, we generated a reference transcriptome in two endemic *Satureja* species of Iran, containing different yields (*Satureja khuzistanica* and *Satureja rechingeri*). Cross-species differential expression analysis was conducted between two species of *Satureja*. 210 and 186 transcripts related to terpenoid backbone biosynthesis were identified for *S*. *khuzistanica* and *S*. *rechingeri*, respectively. 29 differentially expressed genes (DEGs) involved in terpenoid biosynthesis were identified, and these DEGs were significantly enriched in monoterpenoid biosynthesis, diterpenoid biosynthesis, sesquiterpenoid and triterpenoid biosynthesis, carotenoid biosynthesis and ubiquinone and other terpenoid-quinone biosynthesis pathways. Expression patterns of *S*. *khuzistanica* and *S*. *rechingeri* transcripts involved in the terpenoid biosynthetic pathway were evaluated. In addition, we identified 19 differentially expressed transcription factors (such as MYC4, bHLH, and ARF18) that may control terpenoid biosynthesis. We confirmed the altered expression levels of DEGs that encode carvacrol biosynthetic enzymes using quantitative real-time PCR (qRT-PCR). This study is the first report on *de novo* assembly and transcriptome data analysis in *Satureja* which could be useful for an understanding of the main constituents of *Satureja* essential oil and future research in this genus.

## Introduction

Terpenoids are the most abundant compounds of plant secondary metabolites and have been interesting due to their pharmacological utility [1]. The universal precursors of terpenoids consist of IPP (isopentenyl pyrophosphate) and its isomer, DMAPP (dimethylallyl pyrophosphate) [2]. The IPP precursor in plants is produced by two completely separate pathways; mevalonic acid (MVA) and the methylerythritol phosphate (MEP) pathways [3]. The condensation of DMAPP and IPP provides geranyl diphosphate (GPP). The GPP is the immediate precursor to monoterpenes, diterpenes, and tetraterpenoids. They are the main components of many essential oils extensively used as fragrances, cosmetics ingredients, food additives, and the synthesis of perfume chemicals [4].

Carvacrol is a monoterpene compound derived from isoprene hydrocarbure (2-methyl-1, 3-butadiene) and formed by the attachment of two or more isoprene molecules [5]. Carvacrol (over 90%) is the major essential oil produced by Satureja species [6]. It is produced in the essential oils of some members of Lamiaceae family such as Marjoram [7], Fern leaf lavender [8] and Thymes [9].

Satureja (savory) is an important genus belonging to the Lamiaceae family [10], it consists of more than 200 species, including nine species of endemics to Iran. *Satureja khuzistanica* is an aromatic endemic species. This species was first illustrated in 1944 as a new species in the flora of Iran [11]. *S*. *khuzistanica*, as a medicinal herb, is distributed in mountainous slopes in north, northwest, northeast, central and southern parts of Iran [12]. *S*. *rechingeri* is another endemic where it is found only in I1am province, west of Iran [6].

In extensive studies on *Satureja* species, the biological activities [13], including their hypotensive and hypolipidemic [14] effects, antifungal [15], anti-bacterial [16], anti-oxidant [17], anti-parasitic [17], and anti-inflammatory activities [18] have been demonstrated. The importance medicinal of savory has been attributed to the accumulation of carvacrol in essential oil and free phenolic acids, especially rosmarinic acid [19].

Previous studies have focused on the phytochemical analysis [20, 21] and pharmacological activities of savory essential oil [13] while genes involved in the biosynthesis pathway of essential oil compounds are poorly investigated in this plant.

RNA sequencing (RNA-Seq) is a high throughput technique based on next-generation sequencing (NGS) technology [22]. mRNA sequencing can provide transcripts information to examine gene expression levels and can help us to understand more about functional genes in the transcriptome study of organisms without the genomic sequence [23]. RNA-Seq strategy can be used to identify and characterize secondary metabolite genes and their pathways [24, 25]. Transcriptome analysis using RNA-Seq had been applied in medicinal herbs such as *Salvia miltiorrhiza* [26], *Dracocephalum tanguticum* [27], *Cymbopogon flexuosus* [28], *Hoveniaacerba* [29], *Swertia mussotii* [30] and *Piper nigrum* [31].

Despite its medicinal value, knowledge about the genetics of *Satureja* is limited, which has hindered the investigation of metaboliteites synthesis. Therefore, comprehensive research is important to increase the biological understanding of this genus and to clarify the metabolic pathways leading to the biosynthesis of valuable compounds [30].

In the present study, *S*. *khuzistanica* and *S*. *rechingeri* were used as the experimental materials to establish a representative model of transcriptome sequences of *Satureja* using Illumina sequencing. The transcriptome database was used as reference data to identify candidate genes associated with carvacrol biosynthesis. Based on this, we used differential expression profile analysis to compare the transcripts involved in carvacrol biosynthesis. The transcriptome sequences and gene expression profiles provide a database for functional genomic studies on *Satureja* in the future and will facilitate a better understanding of the molecular mechanisms of secondary metabolites biosynthetic pathways.

## Materials and methods

### Plant material

*S*. *khuzistanica* and *S*. *rechingeri* were collected from Khorramabad city, Lorestan province, Iran. In the present study, leaf tissue (the main part of the biosynthesis of secondary metabolites) was used to generate the transcriptome of *Satureja*. The leaves were collected during the full flowering stage (The phase containing the highest essential oil content) [32–34]. Three biological replicates were considered for each species. All samples were snap-frozen in liquid nitrogen and stored at −80°C until used.

### RNA extraction

Total RNA was isolated from all replicates of each species with TRIzol Reagent (Invitrogen, Carlsbad, California, United States), according to the manufacturer’s protocol, and then DNase I (Takara, Japan) was used to eliminate genomic DNA. The RNA quantity and quality were determined with Agilent 2100 Bioanalyzer (Agilent Technologies, USA). A portion of the extracted RNA was sequenced in each biological replication, and the remaining part was used for qRT-PCR analysis.

### RNA-Seq library preparation and Illumina sequencing

The libraries were created with high-quality total RNA samples (OD_260/280_ = 1.8 ~ 2.2; OD_260/230_ ≥ 2.0; RNA Integrity Number (RIN) ≥ 8 out of 10; 28S:18S > 1.0). The cDNA libraries were prepared according to Illumina’s protocol (Illumina Inc., San Diego, USA; cat. no. RS-100-0801) by a genome sequencing company (Novo gene, China) and were sequenced to a depth of 40 million paired-end reads per each biological replicate using an Illumina HiSeq™ 2500 platform (150 bp; Illumina).

The transcriptome raw sequencing data of *S*. *khuzistanica* and *S*. *rechingeri* have been submitted to the NCBI GenBank (https://www.ncbi.nlm.nih.gov) with BioSample accession numbers as following: SAMN09079833, SAMN09079834, SAMN09079835 and SAMN09080197, SAMN09080198, SAMN09080199 and BioProject accession numbers: PRJNA464236, PRJNA464241.

### RNA-Seq data processing and *de novo* assembly

The raw read quality was evaluated using the FastQC software (version 0.11.5) (http://www.bioinformatics.babraham.ac.uk/projects/fastqc/) [35]. High-quality reads were filtered by removing the adaptor and low-quality sequences (Q score < 20) from the raw reads using the Trimmomatic tool (v. 0.36) [36]. High-quality reads were assembled using Trinity de novo assembler (v2.8.5) (http://trinityrnaseq.sourceforge.net/) [37] with default parameters and the k-mer size of 25. The result of assembled sequences as transcripts were filtered through trinity to have FPKM (Fragments per kilobase of transcript sequence per millions base pairs sequenced) more than one value per gene. To validate and assess the *de novo* assembly quality, the reads were mapped to the assembled transcripts using Bowtie 2–2.3.4.1 package with the default parameters [38].

### Functional annotation of assembled transcripts

The Trinotate pipeline (https://trinotate.github.io/) was used for gene functional annotation. All of the assembled transcripts were searched against several databases, including NCBI Non-redundant (NR) (http://www.ncbi.nlm.nih.gov/) and Swiss-Prot [39] through BLASTx with an e-value cut-off of 1e^−5^ and only the best homologue was reported. Open reading frames (ORFs) were predicted using TransDecoder (v5.5.0) (http://transdecoder.sourceforge.net/) following the identification of the longest ORFs. Protein sequences of predicted ORFs were searched using BLASTp against Swiss-Prot and eggNOG database with an e-value cut-off of 1e^−3^, seed_score = 80, and query_cov = 60 [40]. Online KEGG Automatic Annotation Server (v2.1) (KASS) (https://www.genome.jp/kaas-bin/kaas_main?mode=partial) with P-value cut-off of ≤ 0.05 was performed to determine Pathways, KO assignments, and functional descriptions of assembled transcripts [41]. Protein domains were identified by searching against Pfam-A HMMs database with default parameters and e-value cut-off of 1e^−3^ [42].

### Identification of differentially expressed genes (DEGs)

To evaluate the abundance of transcripts in each species, transcripts quantification for each biological replicate was performed by RSEM software [43] and bowtie2 was used for alignment, and then the gene expression matrix was built to generate a normalized expression value. The ExN50 analysis was used to estimate the expression levels of the transcripts. DEGs were identified from the count’s matrix estimated by Integrated Differential Expression Analysis MultiEXperiment (IDEAmex) web server (http://zazil.ibt.unam.mx/idea) with four methods: edgeR, NOISeq, limma, and DESeq2 [44]. The DEGs with log 2-fold change ≥ 2 (up-regulated genes) and ≤ − 2 (down-regulated genes) and P-value cut-off of ≤ 0.01 were screened, and only transcripts that were differentially expressed according to the four methods considered as DEGs. Venny (v2.0) was used to compare the four methods results of DEGs [45]. Web Gene Ontology Annotation Plot (WEGO) was used for Gene Ontology (GO) terms [46] and g:Profiler was used to perform GO and KEGG enrichment analysis (Organism: *Arabidopsis thaliana*, Statistical domain scope: only annotated genes, Significance threshold: g:SCS algorithm, P-value cut-off of < 0.01) [47].

### Identification of genes related to essential oil compounds biosynthesis

Candidate transcripts of secondary metabolites in *S*. *khuzistanica* and *S*. *rechingeri* were identified using BLASTx (length > 80 bp, percentage of identical matches > 70 mismach< 150 and, e-value cut-off of 1e^−5^) against NCBI NR and Swiss-Prot databases and BLASTp (Assignment method: SBH (single-directional best hit), manually annotated organisms: Monocots and Eudicots, P-value cut-off of ≤ 0.05) against KEGG Automatic Annotation Server Ver. 2.1. The Trinotate pipeline was used to assign GO terms to the annotated sequences to predict the functions of the sequences. All of the transcripts related to terpenoid biosynthesis pathways were identified based on the annotation results. Expression values of transcripts involved in the terpenoid backbone biosynthesis and monoterpenoid biosynthesis pathways of *Satureja* were illustrated as a heatmap using HemI software (Heatmap Illustrator (v1.0)) [48]. Enriched GO-terms were visualized with REVIGO with default parameters [49]. All of the assembled transcripts were searched using BLASTx (e-value cut-off of 1e^−5^) against Plant TF Database v5 (PlantTFDB) [50]. TFs were filtered by alignment length > 80 bp, percentage of identical matches > 70, e-value cut-off of 1e^−5^, number of mismatches < 150 bp and pct_hit_len_aligned > 50. The differentially expressed genes associated with terpene biosynthesis was shown as a heat map using the Clustergrammer (Distance: correlation, Linkage type: complete) (https://maayanlab.cloud/clustergrammer/) [51]. Co-expression interactions networks were constructed using the GeneMANIA prediction web server, with default parameters (Organism: *Arabidopsis thaliana*) (https://genemania.org/) [52].

### Quantitative real-time PCR (qRT-PCR) validation

The qRT-PCR technique was used for expression analysis of selected genes of *Satureja* using the real-time PCR detection system (Step One Real-Time PCR System, Applied Biosystems, USA) and TaKaRa SYBR® Green PermixEx TaqTM II. Primers were designed using Primer premier6 software. Specific oligonucleotides for qRT-PCR analysis are listed in the S1 Table. The thermal profile was 95°C for 10 min, followed by 40 cycles of 95°C for 15 s and 60°C for 1 min. Elongation factor1a (EF1a) as the reference gene was used for qRT-PCR normalization [53]. Data were analyzed using the comparative cycle threshold (Ct) method [54, 55]. Two technical replicates were used for each biological replicate. Statistical analyses were performed using ANOVA with IBMSPSS 24.0 (SPSS, Inc., Chicago, IL, United States). A significance level of P-value cut-off of < 0.05 was applied.

### Essential oil extraction

Fresh leaf tissue (200 g) of *S*. *khuzistanica* and *S*. *rechingeri* was air-dried for 7 days. The essential oil of these plants was extracted using Clevenger for 4 hours. The extracted essential oil was dehydrated over Na_2_SO_4_ and then stored in tightly closed, dark vials at 4°C until analysis [56].

#### GC-MS analysis of essential oil

The GC-MS (Gas chromatography-mass spectrometry) analysis was performed with a Thermoquest Finnigan Trace. A fused silica capillary DB-5 column with 30 m long and 0.25 mm diameter was used. The column temperature reached 60°C to 250°C. Nitrogen carrier gas flowed at a rate of 1 ml/min and the transfer-line temperature was 250°C [56].

The identification of essential oil components was performed by comparing their mass with those of the internal reference mass spectra library and confirmed by comparison of their retention indices (RI) with those reported in the literature. Co-injection of essential oils with authentic standards (Sigma–Aldrich, USA) was performed. Quantification of the relative percentage of essential oil constituents was performed by GC-FID peak area normalization considering an equal response factor for the different chemical classes occurring in the essential oils [57]. The Phytochemical analysis was performed in three replicate for each species. Differences were considered statistically significant when the P-value cut-off of < 0.05. Statistical analysis was performed using IBMSPSS 24.0 (SPSS, Inc., Chicago, IL, United States).

## Results

### RNA-Seq library preparation and sequencing

**Fig 1:**
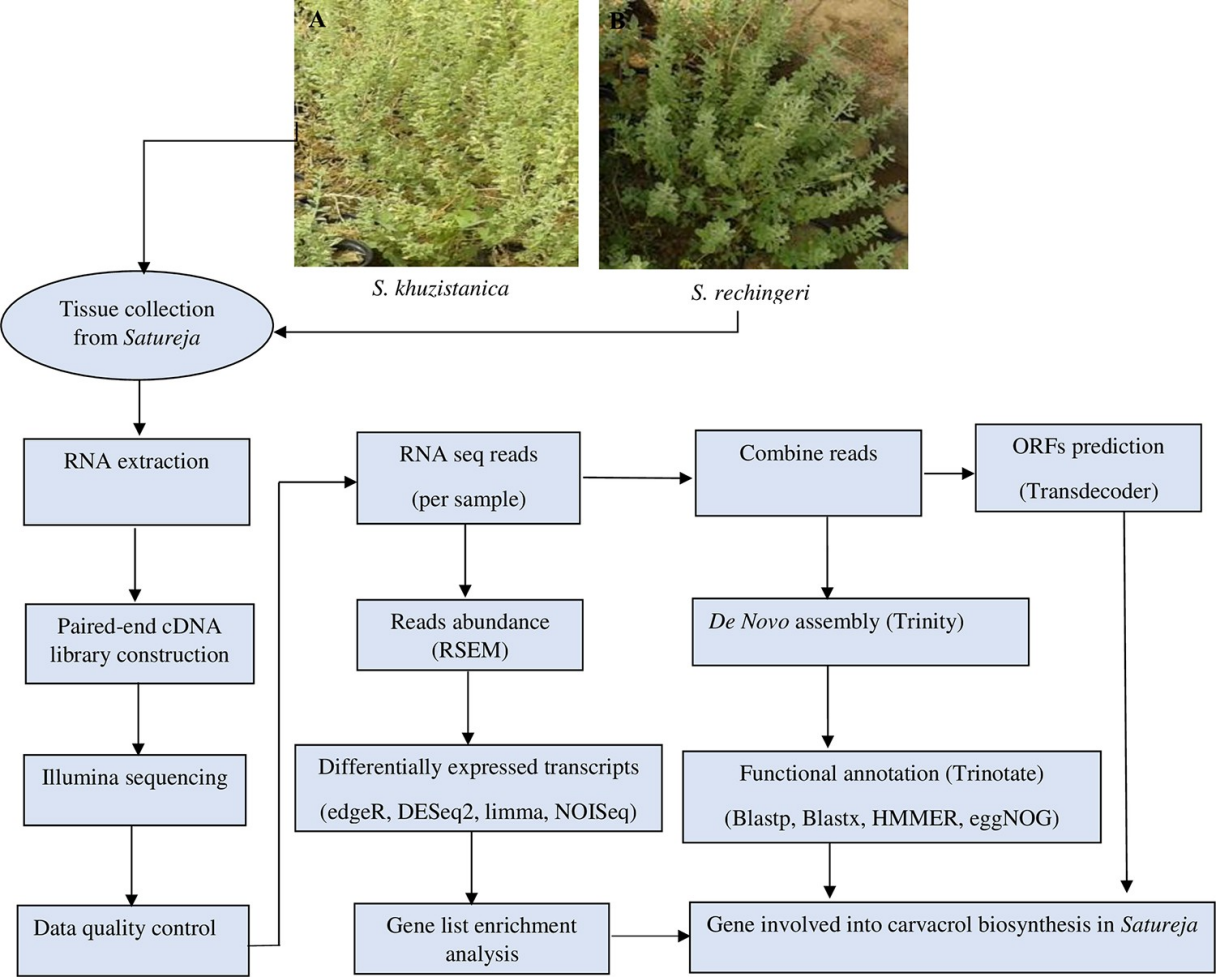
Plant materials. A) *S*. *khuzistanica*, B) *S*. *rechingeri*, Schematic of *de novo* RNA-seq analysis workflow.

Short-read transcriptome sequencing from leaf tissue of *S*. *khuzistanica* and *S*. *rechingeri* was carried out (Fig 1). Over 130 million of 150 bp paired-end reads were acquired for each species. After filtering and deleting poor-quality reads, more than 95% of paired-end reads were considered for downstream analysis (Table 1).

**Table 1:** Number of paired-end raw reads and trim summary per each biological replicate.

Species	Specie ID of each replicate	Raw reads (PE)	Trimmed reads (PE)	Percentage trimmed (%)
***Saturejakhuzistanica***	*Sk_1*	43646790	41761392	95.68
	*Sk_2*	43057192	41444554	96.25
	*Sk_3*	43881896	42512350	96.88
***Saturejarechingeri***	*Sr_1*	44198084	42597116	96.38
	*Sr_2*	44659238	43229780	96.80
	*Sr_3*	45212806	43923756	97.15

#### *De novo* assembly

In the present study, two strategies were considered for *de novo* transcriptome assembly. The first strategy involved one assembled transcript for each biological replicate. For the second strategy, to obtain a more complete assembly, the reads of three replicates of each species were merged (one assembly per species) (S2 Table). The combined assemblies showed higher N50 values than transcripts assembled for each biological replicate. Therefore, the study was performed with data from the second strategy. The transcript assembly details are presented in Table 2. After assembling the reads, the average length of the transcripts in *S*. *khuzistanica* and *S*. *rechingeri* leaf transcriptome was 1079 bp (N50 = 1802 bp) and 1018 bp (N50 = 1740 bp), and the average length of them based on only longest isoform per ‘gene’ was 819 bp (N50 = 1504 bp) and 773 bp (N50 = 1426 bp), respectively (Table 2). According to the mapping results, 98.48% and 98.41% of reads were correctly mapped to the assembled transcripts of *S*. *khuzistanica* and *S*. *rechingeri*, respectively.

**Table 2:** Trinity assembly statistics report of combined assembled transcripts.

Counts of transcripts, etc.					
*Satureja khuzistanica*			*Satureja rechingeri*		
Total trinity ‘genes’	Total trinity ‘transcripts’	Percent GC	Total trinity ‘genes’	Total trinity ‘transcripts’	Percent GC
121192	271907	43.93	119203	225534	45.02
Trinity assembly stats					
Stats	based on all transcript contigs	based on only LONGEST ISOFORM per ‘GENE’	based on all transcript contigs	based on only LONGEST ISOFORM per ‘GENE’	
Contig N10	4274	4032	4225	3910	
Contig N20	3241	2980	3173	2868	
Contig N30	2628	2345	2556	2241	
Contig N40	2173	1893	2108	1795	
Contig N50	1802	1504	1740	1426	
Median contig length	652	418	584	385	
Average contig length	1079.12	818.89	1018.04	772.92	
Total assembled bases	293419043	99242431	229602486	92134478	

### Homology search and functional annotation

The annotation was performed using BLASTx against Swiss-Prot and NCBI NR databases (S3 Table). The results showed that 106255 (59.98%) transcripts for *S*. *Khuzistanica* and 102908 (67.11%) transcripts for *S*. *rechingeri* have at least one significant hit against NCBI Nr database. The number of transcripts with open reading frames in each species was identified using the TransDecoder pipeline (S1 Fig). The top 10 most frequent domain families of *S*. *khuzistanica* and *S*. *rechingeri* were protein kinase followed by PPR_2, Pkinase_Tyr, NB-ARC, p450, LRR_8, RRM_1, WD40, Myb_DNA-binding, and PPR (S2 Fig). The global and overview KEGG pathway maps that detected in *S*. *khuzistanica* and *S*. *rechingeri* is shown in Table 3.

**Table 3:** Global and overview KEGG pathway maps of *S*. *khuzistanica* and *S*. *rechingeri* transcripts defined by KAAS.

KEGG Pathways	(Ko^a^)	NGKP^b^	NIGKPSK^c^	NIGKPSR^d^
**Metabolic pathways**	01100	4121	935	936
**Biosynthesis of secondary metabolites**	01110	1179	425	425
**Microbial metabolism in diverse environments**	01120	1180	155	154
**Biosynthesis of antibiotics**	01130	1016	203	203
**Carbon metabolism**	01200	353	98	98
**2-Oxocarboxylic acid metabolism**	01210	82	29	29
**Fatty acid metabolism**	01212	84	29	31
**Biosynthesis of amino acids**	01230	232	100	100
**Degradation of aromatic compounds**	01220	215	5	6
**Total**	-	8247	1979	1982

^a^Ko: Reference KEGG pathway number

^b^NGKP: Number of genes in each KEGG pathway

^c^NIGKPSK: Number of identified genes in KEGG pathway of *S*. *khuzistanica*

^d^ NIGKPSR: Number of identified genes in KEGG Pathway of *S*. *rechinger*

### Identification of differentially expressed genes (DEGs)

**Fig 2:**
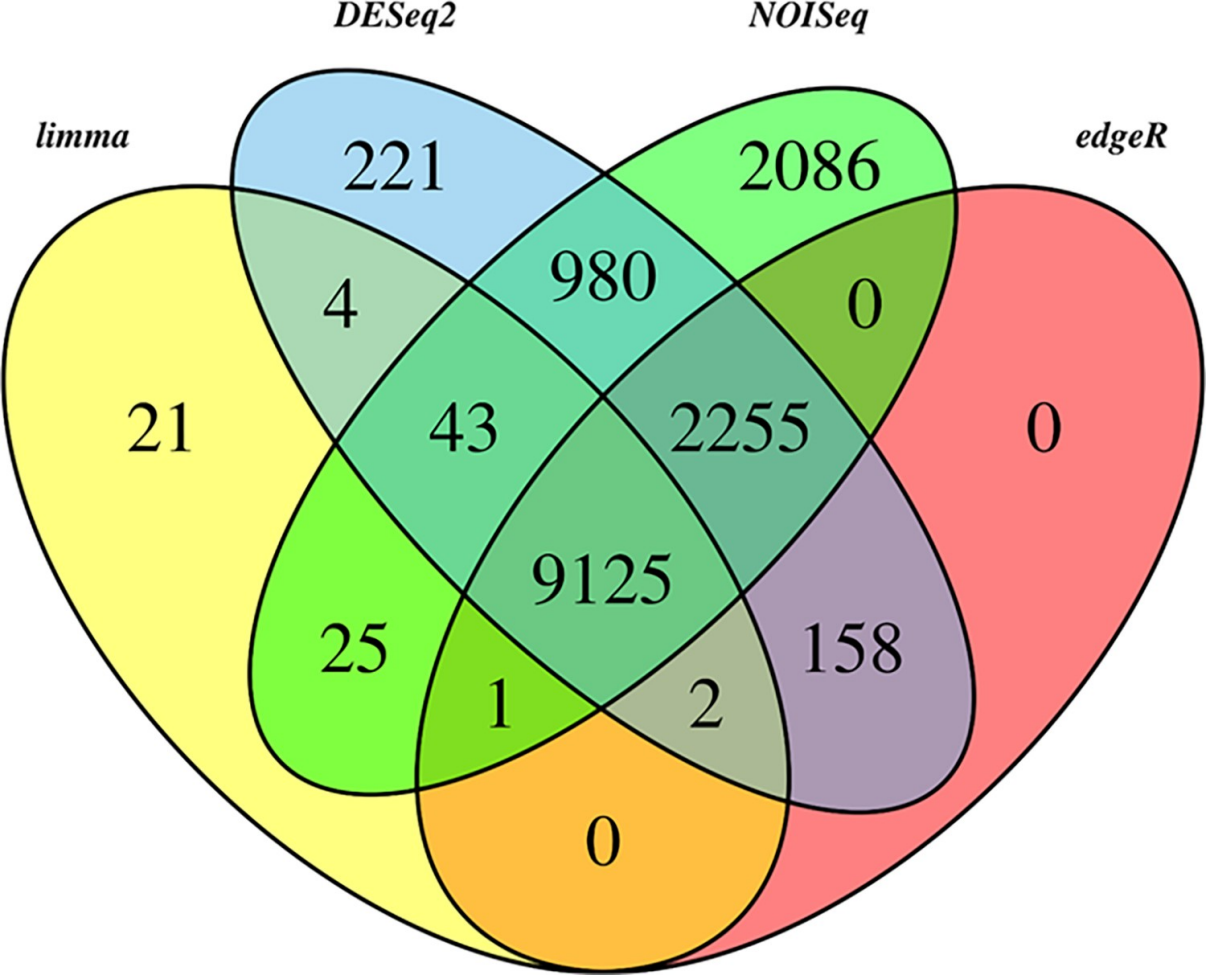
Venn diagram of four statistical methods (edgeR, NOISeq, limma, and DESeq2) of differentially expressed genes in *S*. *khuzistanica* vs *S*. *rechingeri*.

To reduce the number of comparisons, 9125 genes were differentially expressed according to the four statistical methods (edgeR, NOISeq, limma, and DESeq2) and were considered DEGs (Fig 2). Out of 9125 differentially expressed genes between the two species, 7026 transcripts were annotated against Swiss-Prot database (S4 Table). Among these DEGs, 4133 genes were up-regulated and 2893 genes were down-regulated under *S*. *khuzistanica* vs *S*. *rechingeri*.

### GO enrichment analysis for differentially expressed genes

**Fig 3:**
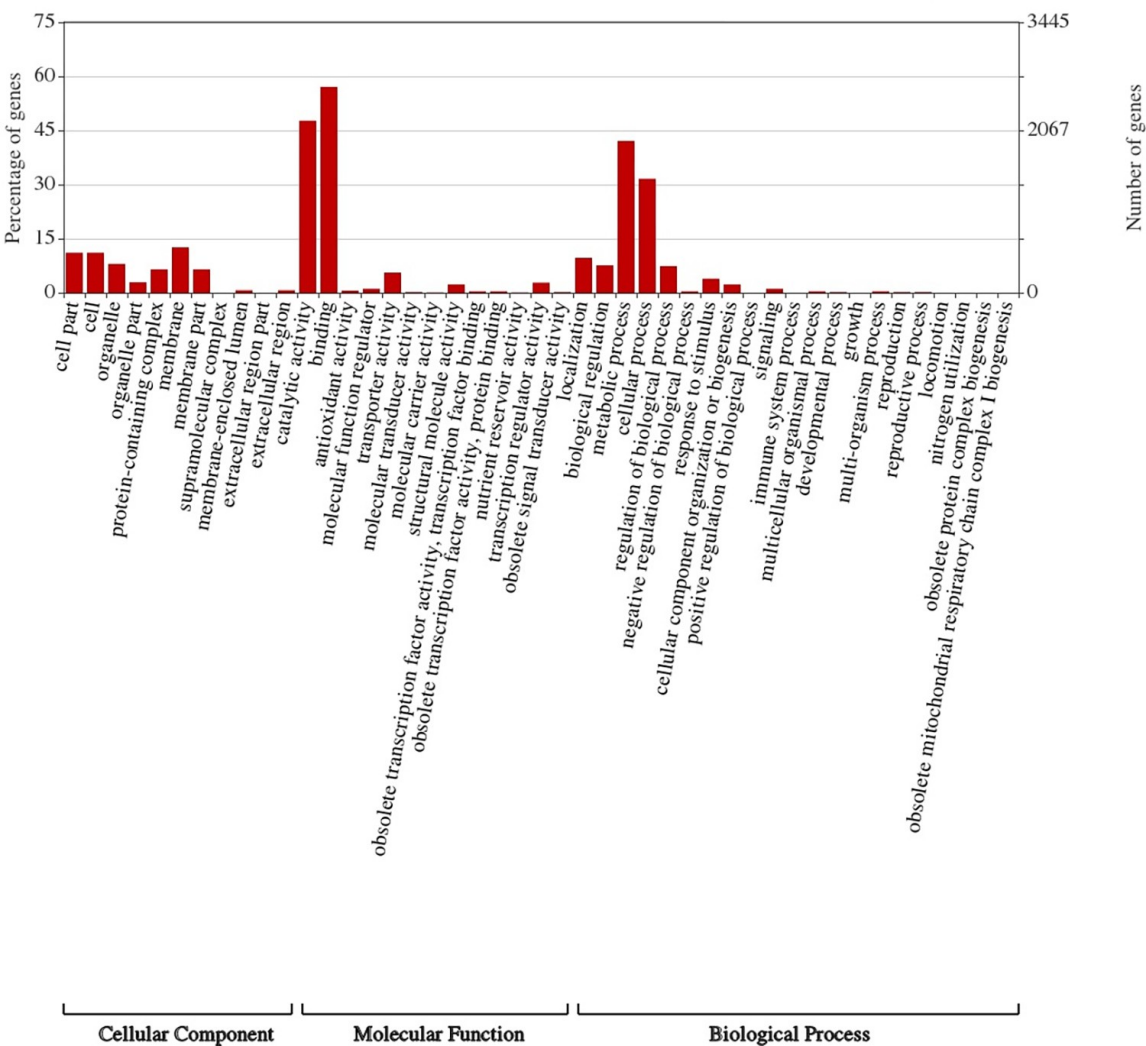
Gene ontology (GO) classification of annotated DEGs. The x-axis indicates the subgroups in GO terms; y-axis shows the percentages of genes (number of a particular gene divided by total gene number).

In total, the 4594 DEGs were assigned to three Gene ontology (GO) terms (biological process, molecular functions, and cellular component) (Fig 3). Most of the DEGs were classified in binding (GO: 0005488) and catalytic activity (GO: 0003824) in the molecular function category. In the biological process category, the majority of the GO terms were related to the metabolic process (GO: 0008152) followed by cellular process (GO:0009987). For the cellular components, the majority of the GO terms were related to membrane (GO: 0016020), and cell part (GO: 0044464) (Fig 3).

**Fig 4:**
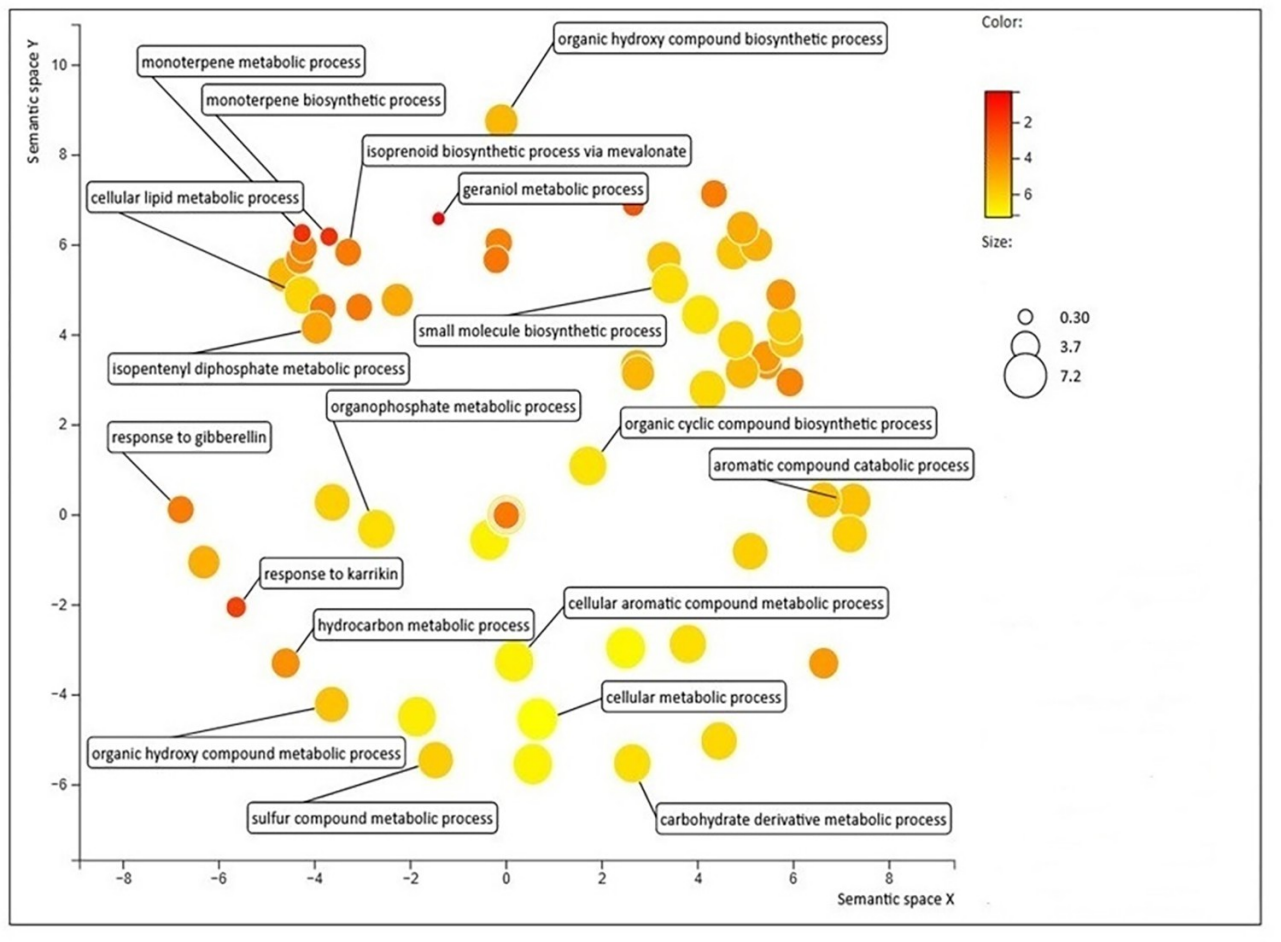
The scatterplot of selected terpenoid biosynthesis view of GO category enrichment analysis of differentially expressed genes related to biological process found in *S*. *khuzistanica* vs *S*. *rechingeri* produced by REVIGO. Circles depicted by filled color show significantly enriched GO terms with log10 p-value <0.05. The color of the bubbles shows the P-value and the size of the bubbles shows the frequency of the GO term in the underlying GOA database.

GO enrichment analysis was done on the DEGs. In all, 1318 GO terms were used for enrichment from two *Satureja* species (S5 Table). In the biological process class, the enriched categories were related to cellular process (GO:0009987), metabolic processes (GO:0008152), organic substance metabolic process (GO:0071704), developmental process (GO:0032502), cellular metabolic process (GO:0044237) and others (S5 Table). The presence of GO categories related to secondary metabolite biosynthetic process (GO:0044550), isopentenyl diphosphate biosynthetic process, mevalonate pathway (GO:0019287), and Acetyl-CoA metabolic process (GO:0006084) led us to identify the genes related to terpenoid biosynthesis in *Satureja* species (S5 Table). GO enrichment was analyzed for DEGs related to the terpenoid biosynthesis in *Satureja* (S6 Table). As a complementary approach to the GO analysis, the scatterplot of the selected terpenoid biosynthesis view of GO category enrichment analysis was constructed based on DEGs related to the biological process of *S*. *khuzistanica* vs *S*. *rechingeri* (Fig 4). the most enriched biological process terms for over-presented DEGs were cellular lipid metabolic process (GO:0044255), organic cyclic compound biosynthetic process (GO:1901362), and cellular aromatic compound metabolic process (GO:0006725). Among all the sets in the biological process, three enriched categories were related to the monoterpene metabolism, GO:0043693 as a monoterpene biosynthetic process, GO:0043692 as a monoterpene metabolic process, and GO:1902767 as an isoprenoid biosynthetic process via mevalonate in *S*. *khuzistanica* vs *S*. *rechingeri* (S6 Table).

### Pathway enrichment analysis for differentially expressed genes

The protein sequence of predicted ORFs of *S*. *khuzistanica* (91467 transcripts) and *S*. *rechingeri* (78733 transcripts) were searched to identify transcripts related to secondary metabolic pathways. The results showed that 44.29% of all transcripts were assigned to 401 KEGG pathways.

**Fig 5:**
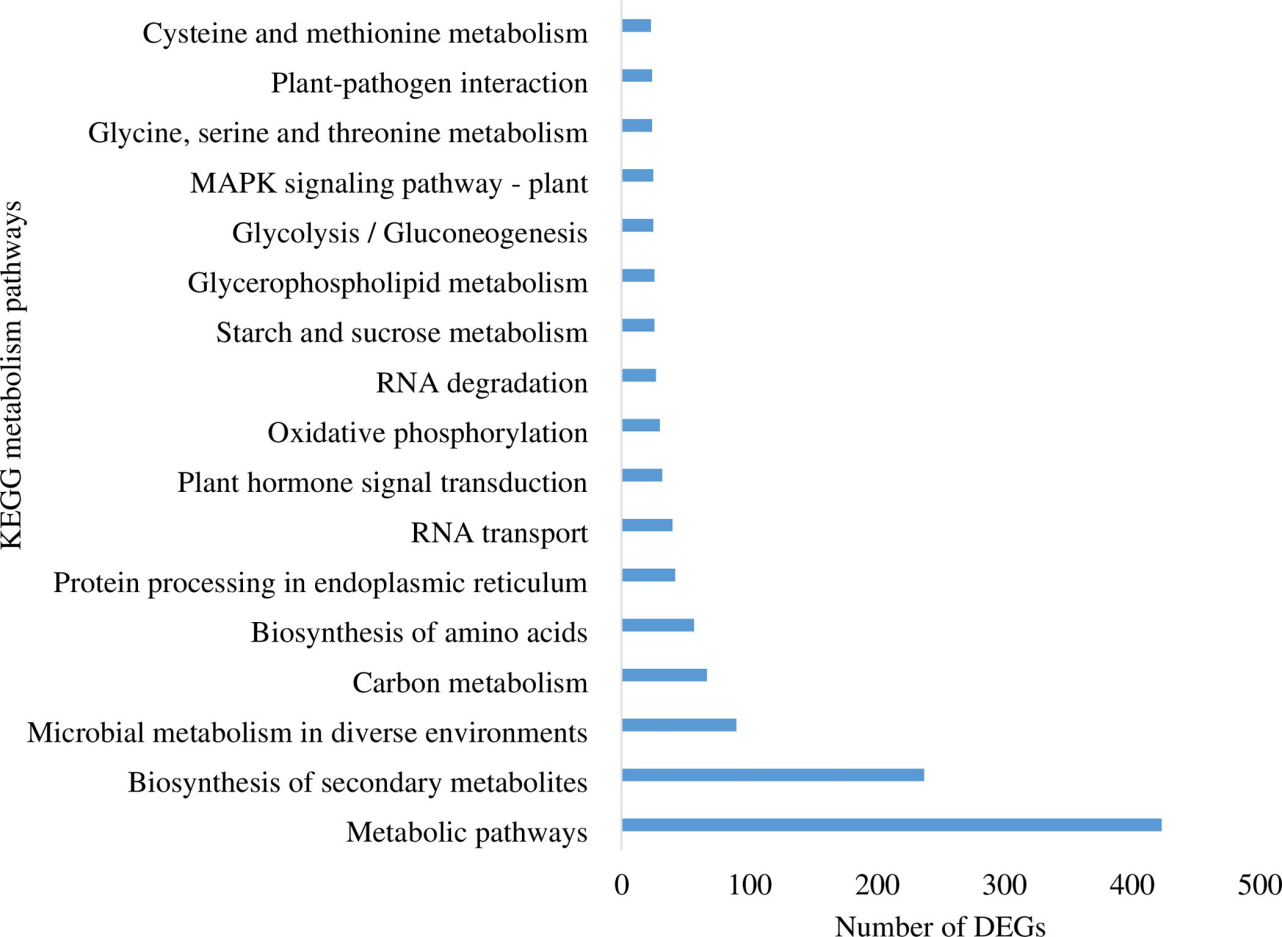
Top hits of KEGG metabolism pathway categories of DEGs.

To identify the biological pathways, DEGs were mapped to KEGG pathways. In all, 4140 DEGs were assigned to 374 KEGG pathways (S7 Table). The highest number of DEGs was assigned to metabolic pathways followed by biosynthesis of secondary metabolites, microbial metabolism in diverse environments, and carbon metabolism (Fig 5). The most common enriched pathways were ‘metabolites biosynthesis’ and ‘biosynthesis of secondary metabolites.

Pathway enrichment analysis for DEGs related to the terpenoid pathway in *Satureja* was used to identify significant KEGG metabolic pathways (Tables 4 and 5). Based on the results of the pathway enrichment analysis, 74 terpenoid biosynthesis-related transcripts were identified (S8 Table). Of KEGG secondary metabolic pathways, most transcripts were assigned to “phenylpropanoid biosynthesis” (ko00940), “terpenoid backbone biosynthesis” (ko00900), and “Flavonoid biosynthesis” (ko00941) in *S*. *khuzistanica* and *S*. *rechingeri* leaf transcriptome (Table 5 and S9 Table).

**Table 4:** KEGG pathway enrichment analysis of differentially expressed genes.

Enriched pathway term	KEGG ID	P-value	Term size^a^	Query size^b^
**Biosynthesis of secondary metabolites**	01110	8.81911E-10	1236	17
**Ubiquinone and other terpenoid-quinone biosynthesis**	00130	4.45951E-09	39	15
**Terpenoid backbone biosynthesis**	00900	4.45951E-09	61	17
**Monoterpenoid biosynthesis**	00902	0.000274219	7	9
**Isoquinoline alkaloid biosynthesis**	00950	0.000999409	22	6
**Phenylalanine metabolism**	00360	0.001892402	33	6
**Tropane, piperidine and pyridine alkaloid biosynthesis**	00960	0.001932288	36	6
**Tyrosine metabolism**	00350	0.002194744	41	6
**Diterpenoid biosynthesis**	00904	0.003544646	21	16
**Phenylalanine, tyrosine and tryptophan biosynthesis**	00400	0.003544646	56	6
**Synthesis and degradation of ketone bodies**	00072	0.005263202	4	4
**Metabolic pathways**	01100	0.006103447	2273	16
**Cysteine and methionine metabolism**	00270	0.011268698	120	6
**Butanoate metabolism**	00650	0.020577188	20	4
**Biosynthesis of cofactors**	01240	0.036374303	250	14
**Valine, leucine and isoleucine degradation**	00280	0.04636249	52	4

^**a**^Term size: all genes of *Arabidopsis thaliana* associated with the given term

^b^Query size: A set of genes as a query.

**Table 5:** Transcripts related to secondary metabolites biosynthesis in *S*. *khuzistanica* and and *S*. *rechingeri* leaf transcriptome.

Category	KEGG pathways	Ko^a^	NGKP^b^	NIGKPSK^c^	NITKPSK^d^	NIGKPSR^e^	NITKPSR^f^
**Metabolism of terpenoids and polyketides**	Terpenoid backbone biosynthesis	00900	51	31	231	31	210
	Monoterpenoid biosynthesis	00902	38	4	131	4	98
	Sesquiterpenoid and triterpenoid biosynthesis	00909	73	8	63	7	40
	Diterpenoid biosynthesis	00904	66	9	114	9	97
	Carotenoid biosynthesis	00906	49	20	151	20	110
	Brassinosteroid biosynthesis	00905	11	8	79	8	66
	Insect hormone biosynthesis	00908	10	1	37	1	32
	Zeatin biosynthesis	00981	18	7	152	7	102
	Limonene and pinene degradation	00903	12	2	39	2	37
	Geraniol degradation	00281	15	2	9	2	13
	Biosynthesis of ansamycins	01051	32	1	16	1	15
	Polyketide sugar unit biosynthesis	00523	58	1	2	1	3
	Biosynthesis of siderophore group nonribosomal peptides	01053	30	1	4	1	2
**Biosynthesis of other secondary metabolites**	Phenylpropanoid biosynthesis	00940	37	22	620	21	544
	Stilbenoid, diarylheptanoid and gingerol biosynthesis	00945	12	5	153	5	153
	Flavonoid biosynthesis	00941	27	14	208	14	206
	Flavone and flavonol biosynthesis	00944	15	3	14	3	13
	Anthocyanin biosynthesis	00942	16	3	8	2	4
	Isoflavonoid biosynthesis	00943	17	3	33	2	21
	Indole alkaloid biosynthesis	00901	30	1	12	1	14
	Isoquinoline alkaloid biosynthesis	00950	62	8	125	8	125
	Tropane, piperidine and pyridine alkaloid biosynthesis	00960	26	9	80	9	67
	Caffeine metabolism	00232	16	3	9	3	11
	Betalain biosynthesis	00965	7	3	21	3	22
	Glucosinolate biosynthesis	00966	20	3	29	3	23
	Monobactam biosynthesis	00261	28	6	30	6	29
	Streptomycin biosynthesis	00521	19	4	45	4	40
	Neomycin, kanamycin and gentamicin biosynthesis	00524	57	1	24	1	18
	Novobiocin biosynthesis	00401	30	2	22	2	23
	Phenazine biosynthesis	00405	16	2	15	2	8
	Prodigiosin biosynthesis	00333	20	3	31	3	34
	Aflatoxin biosynthesis	00254	13	1	3	1	4
	Biosynthesis of secondary metabolites other antibiotics	00998		1	5	1	4
	Biosynthesis of secondary metabolites unclassified	00999		3	59	3	56

^a^Ko, Reference KEGG pathway number

^b^NGKP, Number of genes in each KEGG pathway

^c^NIGKPSK, Number of identified genes in KEGG Pathway of *S*. *khuzistanica*

^d^NITKPSK, Number of identified transcripts in KEGG Pathway of *S*. *khuzistanica*

^e^NIGKPSR, Number of identified genes in KEGG pathway of *S*. *rechingeri*

^f^NITKPSR, Number of identified transcripts in KEGG pathway of *S*. *rechingeri*.

### Identification of genes encoding key enzymes involved in the monoterpenoid biosynthesis pathway

In this study, 210 and 186 transcripts isoforms related to terpenoid backbone biosynthesis were identified for *S*. *khuzistanica* and *S*. *rechingeri*, respectively, which have not been identified in this genus (S10 Table). Transcriptomic analysis to investigate monoterpenoid accumulation patterns in two species of *Satureja* (*S*. *khuzistanica* and *S*. *rechingeri*) discovered 37 transcripts in the mevalonate (MVA) pathway, including nine key enzymes, and 35 transcripts in the methyl erythritol 4-phosphate (MEP) pathway, involving seven key enzymes. It inferred more differences between different species from the same genus.

In the MVA pathway, 3-hydroxy-3-methylglutaryl-coenzyme A reductase (HMGR) as the key enzyme of this pathway contained the highest number of transcripts (*Sk*: 14 transcripts and *Sr*: 9 transcripts) followed by 3-hydroxy-3-methylglutaryl-CoA synthase (HMGS) (*Sk*: 10 transcripts and *Sr*: 7 transcripts).

In the MEP pathway, 4-hydroxy-3-methylbut-2-enyl diphosphate reductase (HDR) (9 transcripts), followed by 1-deoxy-D-xylulose-5-phosphate synthase (DXS) (*Sk*: 8 transcripts and *Sr*: 7 transcripts) as a catalyst enzyme initiates the MEP pathway contained the highest number of transcripts [58] whereas the number of transcripts for 2-C-methyl-D-erythritol 4-phosphate cytidylyltransferase (MCT), 4-diphosphocytidyl-2-C-methyl-D-erythritol kinase (CMK), and 2-C-methyl-D-erythritol 2,4-cyclodiphosphate synthase (MCS) was lower (S10 Table).

**Fig 6:**
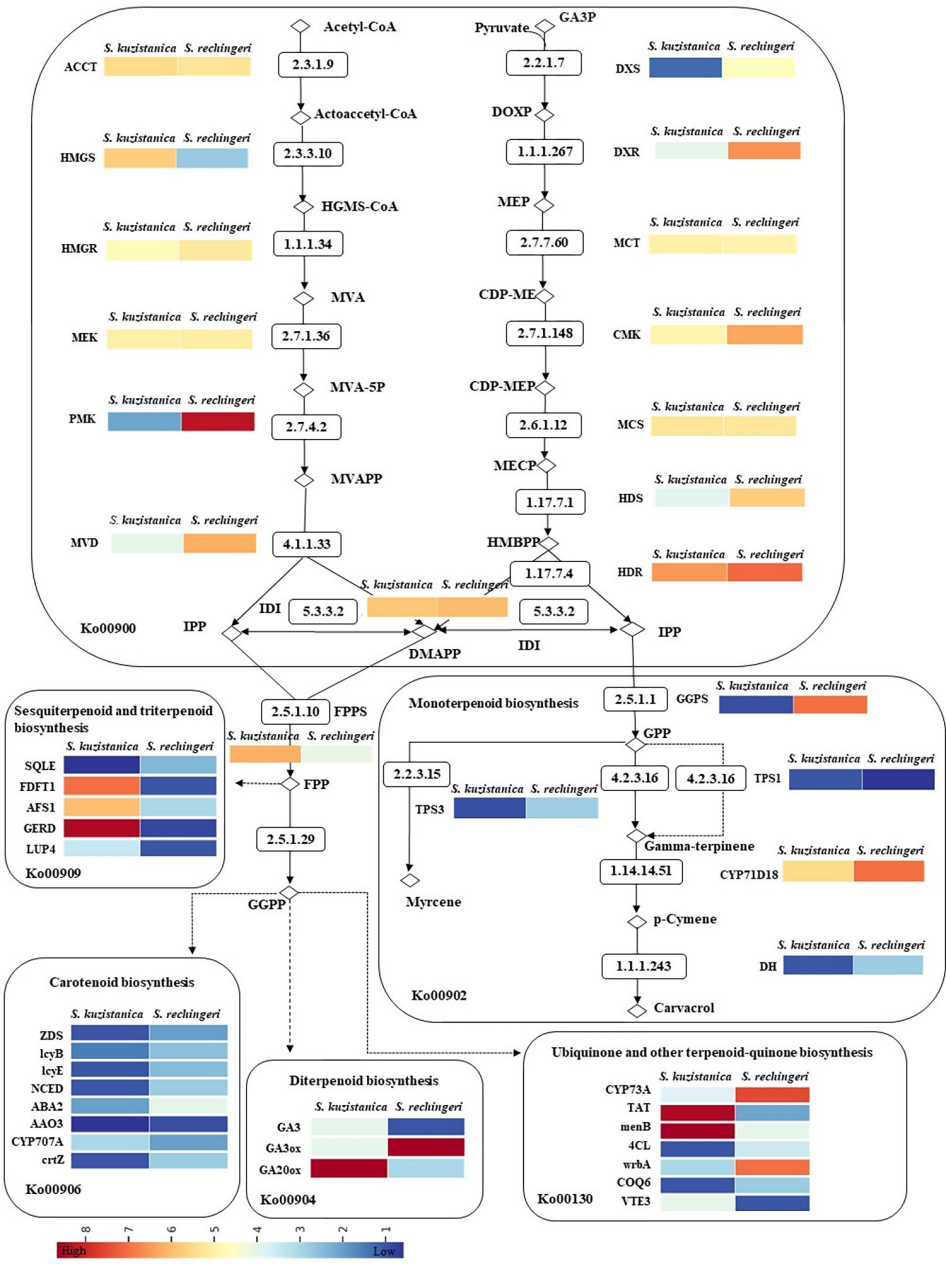
Expression patterns of *S*. *khuzistanica* and *S*. *rechingeri* transcripts involved in the terpenoid biosynthetic pathways. Logarithm of the FPKM value of all transcripts related to each gene was used. Ko numbers show the KEGG maps code related to each pathway. IPP, isopentenyl pyrophosphate; DMAPP, dimethylallyl pyrophosphate; GGPP, geranylgeranyl pyrophosphate; GGPS, GGPP synthase; GPP, geranyl pyrophosphate; FPP, farnesyl pyrophosphate; FPS, FPP synthase; TPS1, terpene synthase 1 (R-linalool synthase); TPS2, terpene synthase 2 ((4S)-limonene synthase), TPS3, terpene synthase 3; DH, carveol dehydrogenase; GA3, ent-kaurene oxidase; GA3ox, gibberellin 3beta-dioxygenase; GA20ox, gibberellin-44 dioxygenase; SQLE, squalene monooxygenase; FDFT1, farnesyl-diphosphate farnesyltransferase; AFS1, alpha-farnesene synthase; GERD, (-)-germacrene D synthase; LUP4; beta-amyrin synthase; ZDS, zeta-carotene desaturase; lcyB, lycopene beta-cyclase; lcyE, lycopene epsilon-cyclase; NCED, 9-cis-epoxycarotenoid dioxygenase; ABA2, xanthoxin dehydrogenase; AAO3, abscisic-aldehyde oxidase; CYP707A, (+)-abscisic acid 8’-hydroxylase; crtZ; beta-carotene 3-hydroxylase; CYP73A, trans-cinnamate 4-monooxygenase; TAT, tyrosine aminotransferase; menB, naphthoate synthase; 4CL, 4-coumarate-CoA ligase; wrbA, NAD(P)H dehydrogenase; COQ6, ubiquinone biosynthesis monooxygenase; VTE3, MPBQ/MSBQ methyltransferase.

We performed differential expression analysis using transcriptome data, to identify candidate DEGs encoding key enzymes in the monoterpenoid biosynthesis pathways. DEGs involved in “Biosynthesis of secondary metabolites” were overexpressed in pair comparisons; DEGs related to terpenoid biosynthetic pathways were detected in *S*. *khuzistanica* vs *S*. *rechingeri* (Fig 6). The 28 key genes related to monoterpenoid biosynthesis, diterpenoid biosynthesis, sesquiterpenoid and triterpenoid biosynthesis, carotenoid biosynthesis, and Ubiquinone and other terpenoid-quinone biosynthesis were discovered by DEGs analysis between *S*. *khuzistanica* vs *S*. *rechingeri*.

As is shown in Fig 6, out of seven genes involved in the MEP biosynthesis pathway, four genes have the highest upregulation in *S*. *rechingeri*. The expression of the DXS gene in *S*. *rechingeri* was double of *S*. *khuzistanica*. The expression of both (E)-4-hydroxy-3-methylbut-2-enyl-diphosphate synthase (HDS) and 1-deoxy-D-xylulose 5-phosphate reducto isomerase (HDR) genes in *S*. *rechingeri* was markedly higher *S*. *rechingeri* compared to that in *S*. *khuzistanica*. The expression of the CMK gene in *S*. *rechingeri* was double of *S*. *khuzistanica*. The expression value of MCT and acetyl-CoA C-acetyltransferase (ACCT) genes was almost equal in both *S*. *khuzistanica* and *S*. *rechingeri*. In the MVA pathway, the expression of the HMGS gene in *S*. *khuzistanica* is higher than in *S*. *rechingeri*, like the HMGR gene. Interestingly, HMGS and phosphomevalonate kinase (PMK) exhibited the highest expression level in *S*. *khuzistanica*.

Secondly, DEGs analyses show that the expression pattern of the core genes encoding enzymes involved in the monoterpene synthases required for carvacrol biosynthesis are expressed at high levels in *S*. *rechingeri* compared to *S*. *khuzistanica*. The expression level of farnesyl pyrophosphate synthase (FPPS) in *S*. *khuzistanica* was higher than that in *S*. *rechingeri*, which was consistent with the expression pattern of DEGs in the MVA pathway. The expression level of geranyl pyrophosphate synthase (GPPS) in *S*. *khuzistanica* was lower than in *S*. *rechingeri*.

Of the 28 key genes, the transcript level of terpene synthases (TPS), Cytochrome P450 71D18 (CYP71D18), and carveol dehydrogenase (DH) genes in the monoterpene biosynthesis pathway were significantly up-regulated in *S*. *rechingeri* vs *S*. *khuzistanica* (Fig 6). The sequence of *Satureja* TPS is more than 80% similar to the known TPS of other plants in the mint family, including TPS2 of *O*. *vulgare* [59] and *Thymus* [60]. CYP71D18 of *Satureja* is more than 80% similar to limonene-3-hydroxylase in *Thymus vulgaris* (S3A and S3B Fig) [60].

Some DEGs were putatively involved in the monoterpenoids biosynthesis pathway (Table 6). The 1279 transcripts were annotated as CYP450s, of which 84 transcripts were differentially expressed in leaf tissues of *Satureja*. The 179 transcripts of TPS gene candidates were identified based on Swiss-Prot database, while 11 transcripts were differentially expressed in the leaf tissue of *Satureja*. The 2309 transcripts that encode the DH genes family were annotated, of which 140 transcripts showed differential expression in leaf tissues of *Satureja*.

**Table 6:** Differentially expressed gene family members which involved in terpenoid biosynthesis in *Satureja*.

Gene family	Differentially expressed genes
Cytochrome P450 (CYP450)	84
Terpene synthase (TPS)	11
Dehydrogenase (DH)	140
Transcription factor (TF)	226

### Analysis of putative transcription factor genes related to terpenoid biosynthesis

Among the DEGs, some important genes were observed such as transcription factors. 226 DEGs associated with TFs gene families in the leaf tissue of *Satureja* were identified (S11 Table) and classified into 41 TF gene families of 34 plant species (S4A and S4B Fig). The top 10 hits of TFs genes families (61.50%, 139 transcripts) were showed by bHLH, MYB_related, NAC, ERF, bZIP, ARF, C3H, HSF, C2H2 and FAR1 (S4A Fig). Among the 41 TF gene families, bHLH (11%, 25 transcripts) had the highest number of transcripts. Among the total species, the highest homology of *S*. *khuzistanica* and *S*. *rechingeri’*s TFs genes were with *S*. *miltiorrhiza’s* TFs genes (91 transcripts, 40%) (S4B Fig).

**Fig 7:**
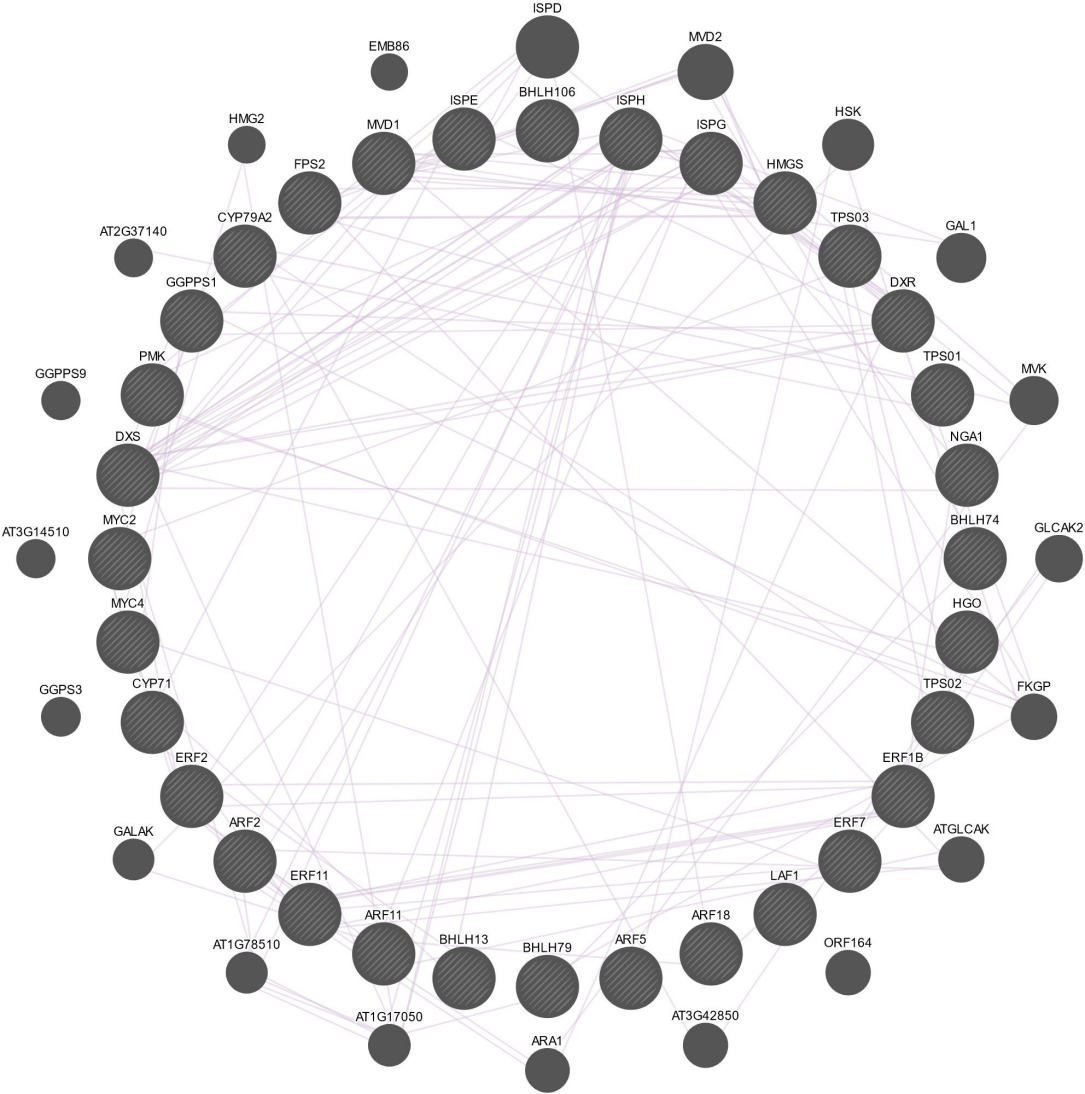
Gene co-expression subnetwork of differentially expressed TFs and terpene biosynthesis-related enzyme genes. Network was reconstructed by GeneMANIA. Connecting lines represent co-expression relationships.

In order to understand the regulatory mechanism of terpenoid accumulation patterns, we analyzed the co-expression profiles between differentially expressed TFs and differentially expressed terpene biosynthesis-related enzyme genes (Fig 7) and their expression pattern as shown in S5 Fig.

As a result, ARF, MYC, bHLH and ERF family genes were predicted to be closely related to terpene biosynthesis genes (Fig 7, S12 Table). The results revealed that HDR was co-expressed with ARF2, ERF2 and bHLH13 genes and HDS was co-expressed with ARF2 and ARF11 genes. MVD was co-expressed with bHLH74. HMGR and TPS3 were co-expressed with MYC2 while CYP71D18 was co-expressed with bHLH13 gene. GGPS was only co-expressed with MYC4. These results highlighted a large number of candidate genes that regulate the accumulation of terpenoids in *Saturej*a.

#### Analysis of essential oil components

Essential oils from leaves of *S*. *khuzistanica* and *S*. *rechingeri* were analyzed (S13 Table). A total of 24 compounds were identified in the essential oil of *S*. *khuzistanica* (sum of approximately 98.96%) and *S*. *rechingeri* (sum of approximately 98.18%). Phytochemical analyses of the leaf tissue showed differences in the amount of the main components of the essential oils. It can be due to the terpene synthase, other secondary metabolite, and their regulator genes (S14 Table).

**Fig 8:**
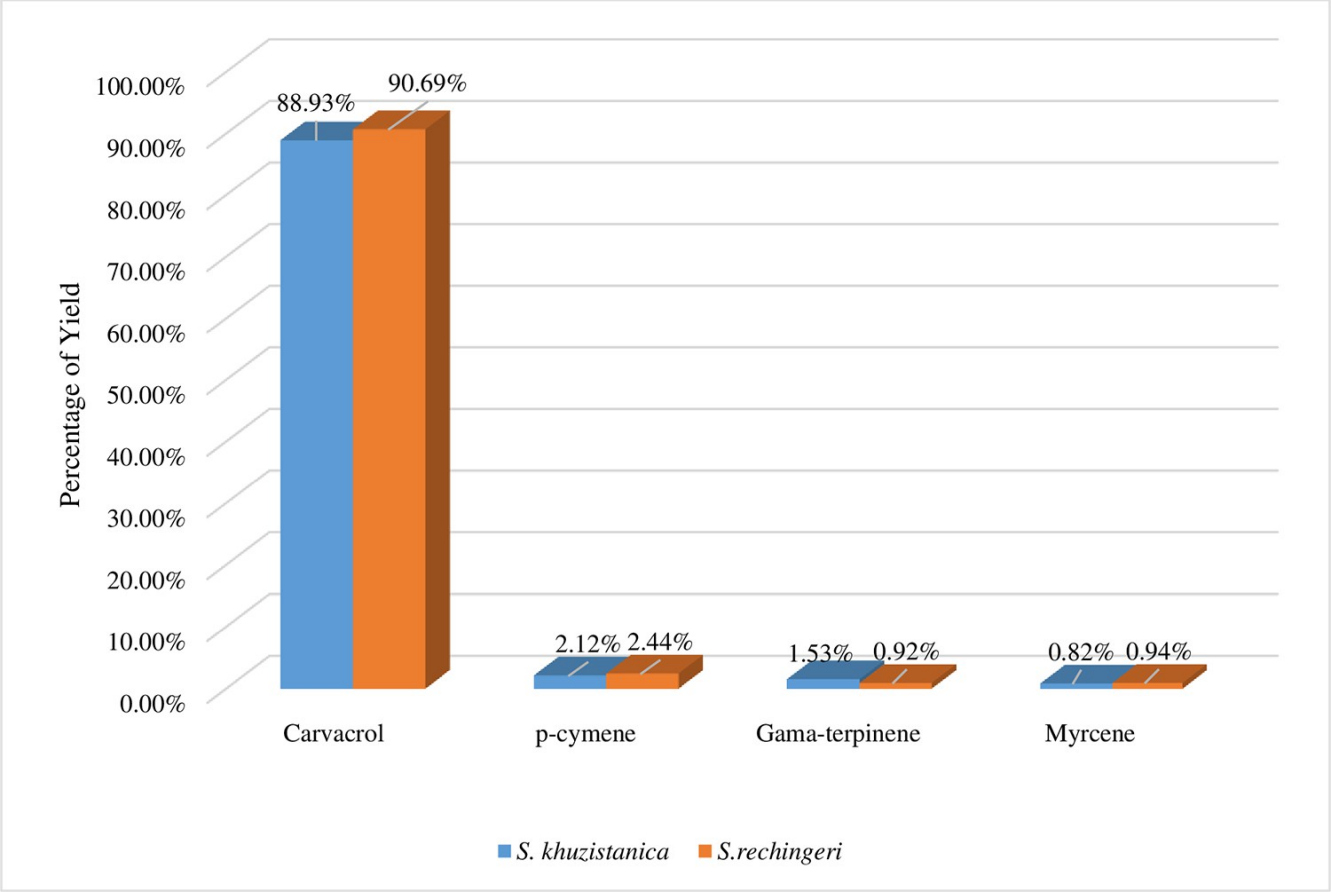
Yield percentage of essential oil’s main components of *S*. *khuzistanica* and *S*. *rechingeri*.

Essential oil profiling by GC analysis represented that hydrocarbon monoterpenes with carvacrol had the highest percentage, followed by p-cymene γ-terpinene (Fig 8), and the percentage of other compounds (borneol, alpha-humulene, and bete-bisabolene) was very low. Also, the results of the variance analysis of the data showed that the difference between the two species of savory is significant at the level of 5% (Table 7).

**Table 7:** The pairwise comparison for the main essential oil components of *Satureja*.

Main component	(I) Species	(J) Species	Mean Difference (I-J)	Std. Error	Sig.b	95% confidence interval for difference	
						Lower Bound	Upper Bound
**Carvacrol**	*S*. *rechingeri*	*S*. *khuzistanica*	3.360*	0.885	0.019	0.903	5.817
**Myrcene**	*S*. *rechingeri*	*S*. *khuzistanica*	0.13	0.12	0.339	-0.202	0.462
**p-cymene**	*S*. *rechingeri*	*S*. *khuzistanica*	1.000*	0.233	0.013	0.353	1.647
**Gama-terpinene**	*S*. *rechingeri*	*S*. *khuzistanica*	0.605*	0.16	0.019	0.16	1.049

*Values are significant at P-value cut-off of < 0.05.

### Verification of RNA-Seq data by qRT-PCR

**Fig 9:**
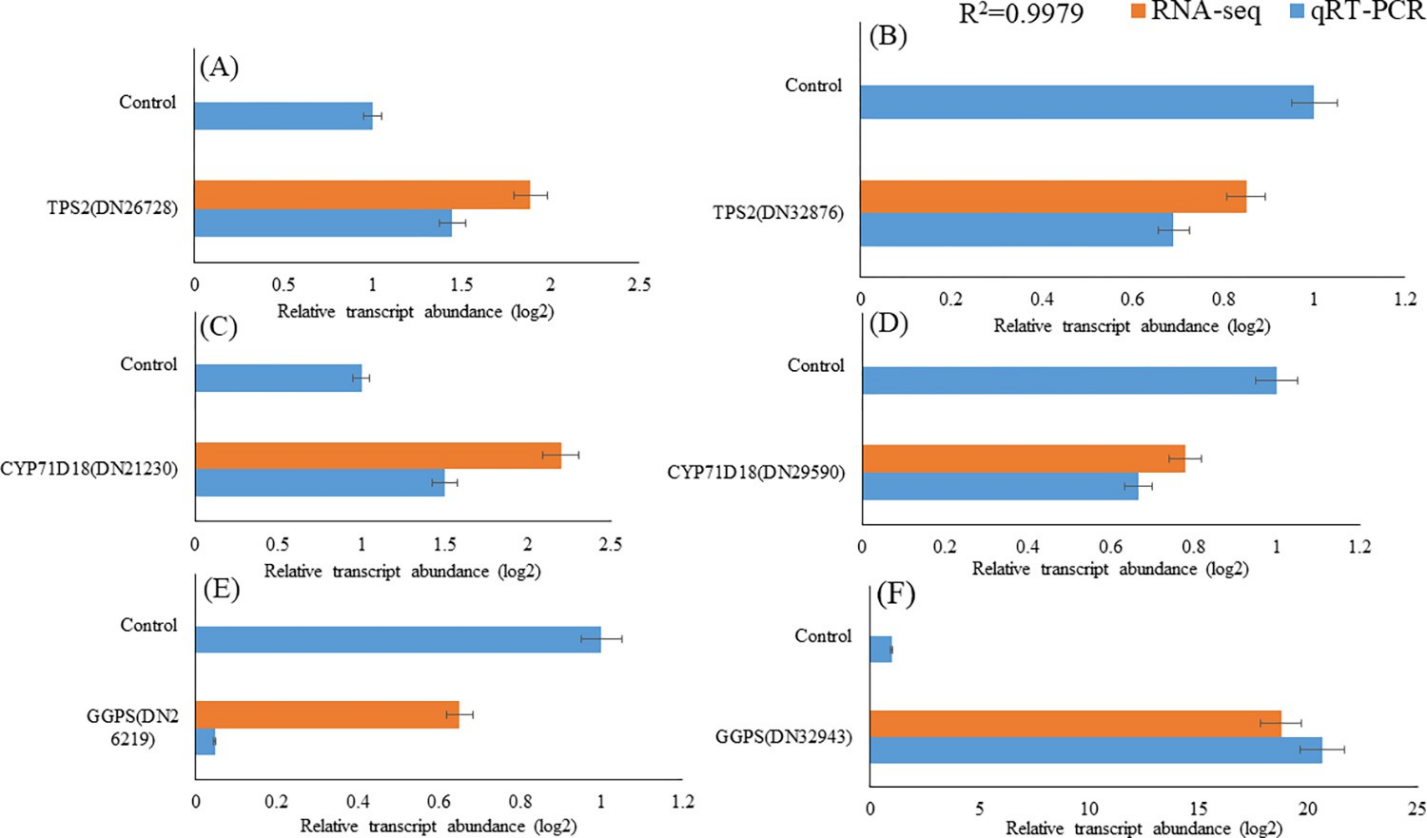
Validation of selected genes using qRT-PCR. (A) TPS2 (DN26728), terpene synthase 2 (B) TPS2 (DN32876), terpene synthase 2 (C) CYP71D18 (DN21230), cytochrome P450 71D18 (D) CYP71D18 (DN29590), cytochrome P450 71D18 (E) GGPS (DN26219), GGPP synthase (F) GGPS (DN32943), GGPP synthase.

In this study, since carvacrol is a major compound of *Satureja* essential oil, the genes involved in this pathway have received more attention. The expression patterns of six DEGs involved in the carvacrol biosynthesis in *Satureja* were validated by using qRT-PCR. As it is shown in Fig 9, the expression value of all six genes is highly consistent and in harmony with their expression from the RNA-Seq results (R^2^ = 0.99). It is important to consider that the slight difference in obtained expression values from the qRT-PCR method with the RNA-Seq results is little due to different parameters such as the primers efficiency, quality of the PCR kit, and the integrity of the thermocycler.

## Discussion

### *De novo* transcriptome assembly

In this study, we report the first analysis of transcriptome data from the *Satureja* genus. The transcriptomes N50 length was higher than that of other members of this family such as *S*. *miltiorrhiza* (1340 bp) [61] and *D*. *tanguticum* (1118 bp) [27]. The annotation result indicated that among the total species, the high homology of *S*. *khuzistanica* and *S*. *rechingeri’*s assembled transcripts with *S*. *miltiorrhiza*; this could be due to the similarity of their family (Lamiaceae) [62].

### The expression of genes involved in the terpenes biosynthesis pathway

To investigate the biosynthetic route leading to carvacrol, we first identified early step genes encoding key enzymes of MVA and MEP pathways. The MEP pathway played a major role in the carvacrol biosynthesis of *Satureja* leaf tissue [63]. As it has shown in Fig 6, genes involved in the MEP pathway show the highest upregulations in *S*. *rechingeri*, with regards to that, the differences in essential oil contents between *S*. *khuzistanica* and *S*. *rechingeri* could have resulted from these genes. The GO results also confirm the presence of more genes related to the MEP pathway of *S*. *rechingeri* than in *S*. *khuzistanica*. The present study suggested that the genes involved in the MEP pathway showed higher expression than the genes involved in the MVA pathway in both species. Both the MVA and MEP pathways were involved in carvacrol production, while the MEP pathway probably played a major role in the biosynthesis of carvacrol [63]. This proposed pathway has some similarities to that of carvacrol biosynthesis in oregano and thyme, in which the carvacrol was biosynthesized from IPP and DMADP which were derived via the MEP pathway [64].

### The expression of genes involved in the monoterpene carvacrol biosynthesis pathway

The gene families of terpene synthases (TPS), cytochrome P450s (CYP450s), dehydrogenase (DHs), and transcription factors (TFs) may be involved in the biosynthesis of carvacrol [65]. The presence of TPS, CYP71D18, HDS, and GPPS genes in the DEG list, might be one of the causes of the increase in the carvacrol amount in *S*. *rechingeri* (S4 Table). In addition, an oregano *OvTPS2* gene encoding a protein with 91% amino acid identity to *Satureja* TPS2 appeared to be highly expressed in tissues producing high amounts of carvacrol [59]. Previous studies showed that the terpene synthase genes of *O*. *vulgare* appear to play a significant role in controlling terpene compounds. This suggests that terpene synthase expression levels directly control the compounds of the essential oil [60]. The TPS2 is mainly responsible for catalyzing the biosynthesis of monoterpenoids [66]. Similarly, *TvTPS2* transcript levels correlated positively with the high-carvacrol and -thymol levels in *Thymus* [67]. The terpene synthase genes can be utilized as markers for the breeding of medicinal plants with higher-value essential oil [68]. P450 family members are very important into the production of terpenoid secondary metabolites (monoterpenoids, diterpenoids, triterpenoids, and sesquiterpenoids) in plants [69]. The role of CYP450 family members in the biosynthesis of secondary metabolites compounds in medicinal plants such as *S*. *miltiorrhiza* [70], *Taxus chinensis* [71], *Catharanthus roseus* [72], *Medicago truncatula* [73] and *Panax ginseng* [74] has also been mentioned. Transcriptomic analysis of *Satureja* lead to the identification of CYP71D18, which exhibits 90.1% amino acid identity with *OvCYP71D181* and was well correlated with carvacrol levels. In the study of Crocoll et al. (2010) on the *O*. *vulgare* L., the role and involvement of CYP71D180 and CYP71D181 in the production of thymol and carvacrol were reported [60]. In *Mentha*, the role of P450 monooxygenases was confirmed in the biosynthesis of menthol and carvacrol [75].

### Terpenoid biosynthetic regulation

One of the most important ways to control gene expression is transcriptional regulation that TFs play a critical role in it [76]. They regulate diverse biological processes such as growth, development, and secondary metabolism [77]. Several members of AP2/ERF, bHLH, MYB, WRKY [76,78], NAC, DOF, bZIP, and HD-ZIP family proteins have been reported as regulators of secondary metabolite biosynthesis [79–81]. A positive correlation between the expression of differentially expressed TFs and the levels of differentially expressed terpene biosynthesis-related enzyme genes was also found. It has become evident that Both the MVA and MEP pathways are heavily regulated [82]. Basic bHLH TFs play a pivotal role in the biosynthesis of secondary metabolites [83]. MYC family members are bHLH TFs [84]. Some MYC TFs control terpenoid biosynthesis in plants [78]. In lavender, *LaMYC4* was involved in the regulation of terpenoids and affects the expression of HMGR, DXS, DXR, FPPS, and GPPS in the terpenoid synthesis pathway. In addition, DXR expression was strongly associated with the expression of *LaMYC4* [85]. Previous transcriptome studies highlighted the close relationship between the silencing of NAC2 and the up-regulation of the expression levels of HMGR and DXS [86]. HMGR and DXS played important roles in the regulation of MVA and MEP pathways, respectively [87, 88].

### Phytochemical analyses

Phytochemical analyses of the leaf tissue showed carvacrol content in *S*. *rechingeri* increased vs *S*. *khuzistanica*. Gama-terpinene, the second important compound of the savory plant, showed a significant difference between the two species. This is in agreement with the previous studies of essential oil components [56]. The regulation pattern of genes involved in terpenoids biosynthesis is probably due to the different genetic backgrounds of the two species. This data corresponded to the higher content of monoterpenoid in *S*. *rechingeri* and was confirmed by qRT-PCR.

Taken together, the results of previous studies confirm that the difference in the expression level of genes involved in terpenoid biosynthesis and identified transcription factors can be the cause of might be one of the causes of changing in the secondary metabolite biosynthesis amount in *S*. *khuzistanica* and *S*. *rechingeri*.

## Conclusion

This study reported the results of comparative transcriptome analysis and key genes for investigation of the production of monoterpenoid in *Satureja*. A total of 60% of transcripts from all the libraries were annotated. Two species were evaluated for essential oil composition. Several transcripts encoding key enzymes involved in the MVA and MEP biosynthetic pathways were identified, which produce precursors for carvacrol biosynthesis. In total, 7026 DEGs were expressed in the different pattern in *S*. *rechingeri* compared to *S*. *khuzistanica*. A total of 461 DEGs involved in terpenoid biosynthesis were identified. The expression pattern of transcripts involved in the monoterpene biosynthetic pathway was evaluated. The expression pattern of 19 differentially expressed TFs genes was identified as pathway regulatory genes. These data will facilitate further study of the molecular mechanisms underlying monoterpenoid biosynthesis and gives rise to valuable resource data to explore carvacrol biosynthesis in the future.

## Supporting information

S1 Fig The number of transcripts with open reading frames in each species identified using the TransDecoder pipeline. Complete ORF sequences in which the first codon and the stop codon are present; 5 prime: sequences that contain the start codon but lack the stop codon; 3 prime: partial ORF sequences that lack the start codon; internal: sequences that lack both the start and stop codons.
(TIF)

S2 Fig Top 10 hits of *S*. *khuzistanica and S*. *rechingeri’s* domain families. (TIF)

S3 Fig Phylogenetic analysis of Cytochrome P450 71D181 and terpene synthesis from *Satureja*. A) Cytochrome P450 71D181. The full name of the sequences are *SkCYP71D181*, Cytochrome P450 71D181 [*Saturejakhuzistanica*]; *SrCYP71D181*, Cytochrome P450 71D181 [*Satureja rechingeri*]; *TvL3H*, limonene-3-hydroxylase [*Thymus vulgaris*]; *TvL6H*, limonene-6-hydroxylase [*Thymus vulgaris*]; *MgCYP71D18*, (-)-(4S)-Limonene-6-hydroxylase [*Mentha x gracilis*]; *MgCYP71D94*, Cytochrome P450 71D94 [*Mentha x gracilis*]; *McL6H*, limonene-6-hydroxylase [*Mentha canadensis*]; *Mp(-)-L6H*, (-)-limonene 6-hydroxylase-like cytochrome p450-dependent oxygenase [*Mentha x piperita*], *MgCYP71D95*, 71D95: (-)-(4S)-Limonene-3-hydroxylase [*Mentha x gracilis*]; *MsCYP71D95*, 71D95: Limonene-3-hydroxylase [*Mentha spicata*]; *MpCYP71D15*, (-)-(4S)-Limonene-3-hydroxylase; AltName: Full = Cytochrome P450 isoform PM2 [*Mentha x piperita*]; *McL3H*, (-)P450 limonene-3-hydroxylase [*Mentha canadensis*]; *MaLH*, limonene hydroxylase [*Mentha arvensis*]; *Ms(-)-L3H*, (-)-limonene 3-hydroxylase-like cytochrome p450-dependent oxygenase [*Mentha spicata*], *MpCYP71D13*, Cytochrome P450 71D13; AltName: Full = (-)-(4S)-Limonene-3-hydroxylase; *SiCYP71D95-like*, P450 71D95-like [*Sesamum indicum*]; *SmCYP71D374*; cytochrome P450 [*Salvia miltiorrhiza*] B) terpene synthesis.The full name of the sequences are *TmTPS*, gamma-terpinene synthase [*Thymus migricus*]; *TtTPS* gamma-terpinene synthase [*Thymus trautvetteri*]; *TpTPS*, gamma-terpinene synthase [*Thymus pubescens*]; *TsTPS*, putative gamma-terpinene synthase [*Thymus serpyllum*]; *TdTPS*, gamma-terpinene synthase [*Thymus daenensis*]; *TvTPS2*, terpene synthase 2 [*Thymus vulgaris*]; *TcTPS*, gamma-terpinene synthase [*Thymus caespititius*]; *TfTPS*, gamma-terpinene synthase [*Thymus fedtschenkoi*]; *TkTPS*, gamma-terpinene synthase [*Thymus kotschyanus*]; *OvTPS2*, terpene synthase 2 [*O*. *vulgare*]; *SkTPS2*, terpinene synthase [*Satureja khuzistanica*]; *SrTPS2*, terpinene synthase, [*Satureja rechingeri*]; *OvTPS5*, terpene synthase 5 [*O*. *vulgare*]; *PvTPS10*, terpene synthase 10 [*Prunella vulgaris*]; *SrPS*, pinene synthase [*Salvia rosmarinus*] The evolutionary history was inferred using the Neighbor-Joining method. Evolutionary analyses were conducted in MEGA7. The bootstrap replications were set to 100.
(TIF)

S4 Fig Transcription factor genes of *Satureja*. A) Percent of identified TFs families B) Percent of *Satureja* TFs genes with high homology of plant species.
(TIF)

S5 Fig Heatmap of differentially expressed TFs families. Average of FPKM of all transcripts related to each TF gene was used. Ko numbers show the KEGG maps code related to monoterpene biosynthetic pathway. Specie ID of each replicate is Sk_1: *S*. *khuzistanica*_1, Sk_2: *S*. *khuzistanica*_2, Sk_3: *S*. *khuzistanica*_3, Sr_1: *S*. *rechingeri*_1, Sr_2: *S*. *rechingeri* _2, Sr_3: *S*. *rechingeri* _3.
(TIF)

S1 Table Specific oligonucleotides for qRT-PCR analysis. (XLSX)

S2 Table Trinity assembly stats report of assembled contigs. (XLSX)

S3 Table Summary of functional annotation result. (XLSX)

S4 Table Differentially expressed and annotation of total transcripts in *S*. *kuzistanica* and *S*. *rechingeri*. (XLSX)

S5 Table GO enrichment analysis for DEGs transcripts in *Satureja*. (XLSX)

S6 Table GO enrichment analysis for DEGs transcripts related to the terpenoid biosynthesis in *Satureja*. (XLSX)

S7 Table Number of DEGs were assigned to KEGG pathways. (XLSX)

S8 Table DEGs encoding enzymes involved in terpenoid biosynthesis in *Satureja*. (XLSX)

S9 Table Transcripts related to biosynthesis of other secondary metabolites in *S*. *khuzistanica* and *S*. *rechingeri* leaf transcriptome defined by KAAS. (XLSX)

S10 Table Transcripts related to terpenoid backbone biosynthesis in *S*. *khuzistanica* and *S*. *rechingeri* leaf transcriptome defined by KAAS. (XLSX)

S11 Table Differentially expressed and annotation for transcription factor gene families of *Satureja*. (XLSX)

S12 Table Co-expression network among differentially expressed TFs and terpene biosynthesis-related enzyme genes. (XLSX)

S13 Table GC profile of *S*. *khuzistanica* and *S*. *rechingeri* leaf essential oils. (XLSX)

S14 Table The two-way ANOVA for the essential oil components experiment of the leaf tissue of *Satureja* (complete model). (XLSX)
