# Supplementary figures and images for: Comparative transcriptome analysis to identify putative genes involved in carvacrol biosynthesis pathway in two species of Satureja, endemic medicinal herbs of Iran
Source: PLoS One. 2023 Jul 7;18(7):e0281351. doi: 10.1371/journal.pone.0281351 (PMC10328369; doi:10.1371/journal.pone.0281351)

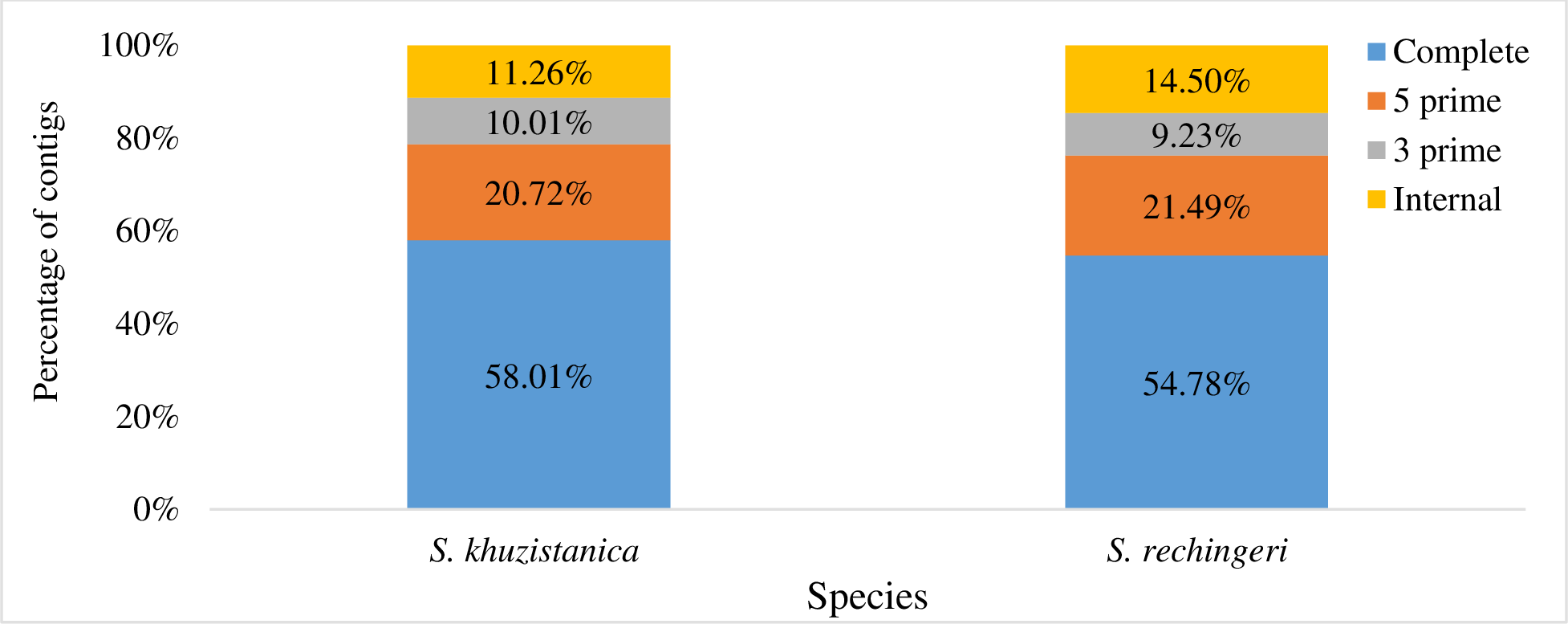

Supplement: S1 Fig — Complete ORF sequences in which the first codon and the stop codon are present; 5 prime: sequences that contain the start codon but lack the stop codon; 3 prime: partial ORF sequences that lack the start codon; internal: sequences that lack both the start and stop codons. (TIF) [file pone.0281351.s001.tif]

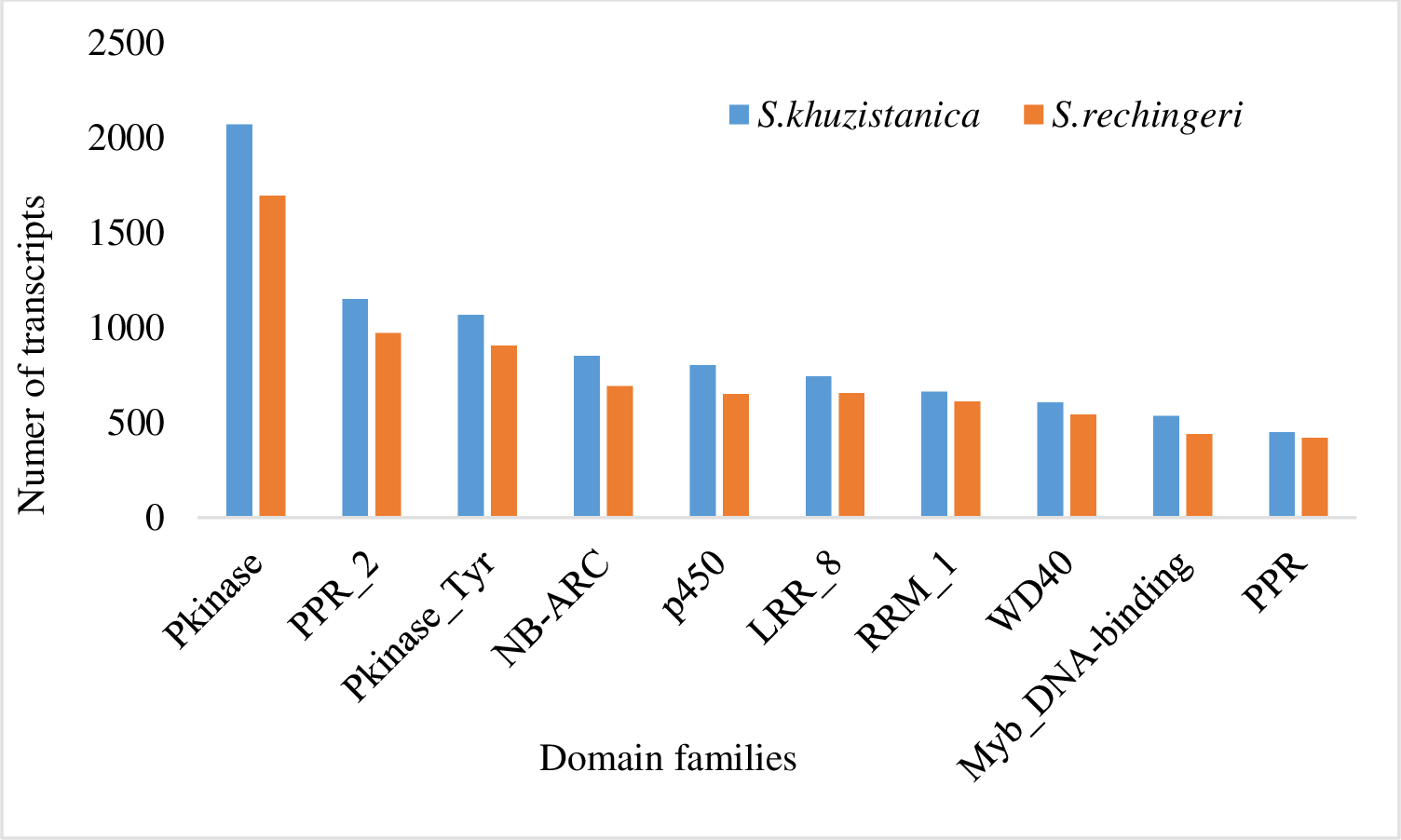

Supplement: S2 Fig — (TIF) [file pone.0281351.s002.tif]

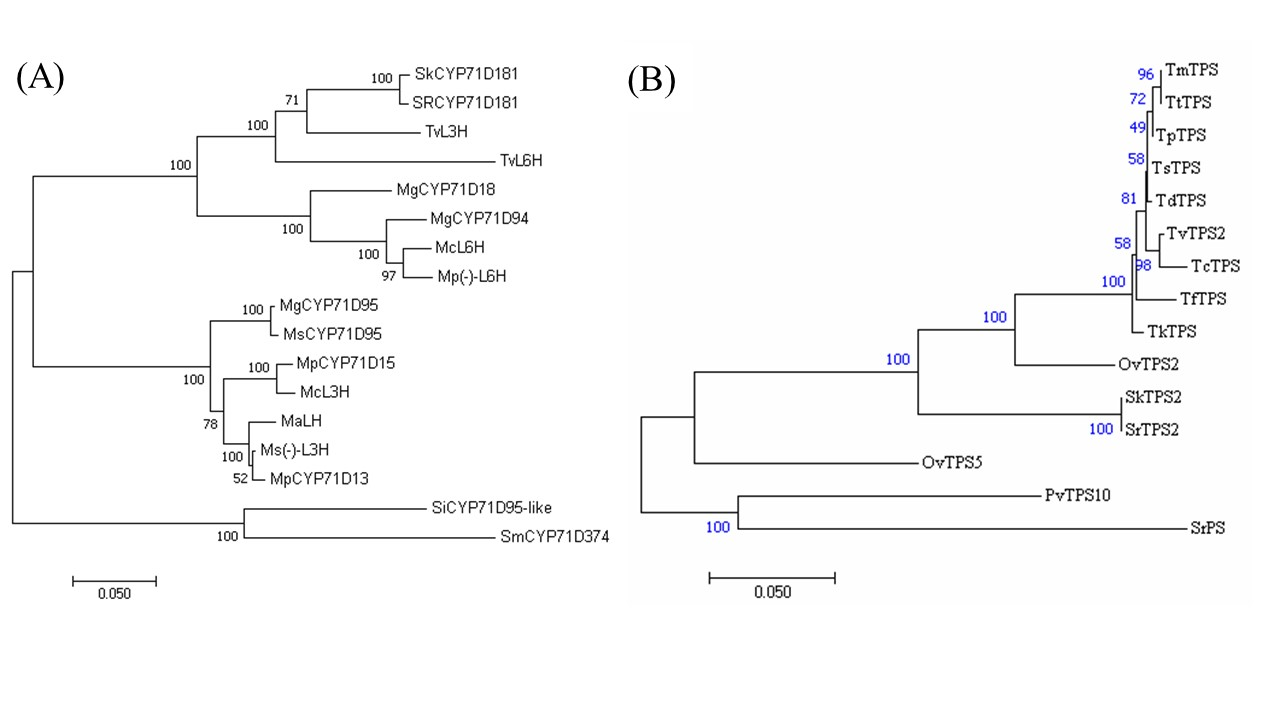

Supplement: S3 Fig — A) Cytochrome P450 71D181. The full name of the sequences are SkCYP71D181, Cytochrome P450 71D181 [Saturejakhuzistanica]; SrCYP71D181, Cytochrome P450 71D181 [Satureja rechingeri]; TvL3H, limonene-3-hydroxylase [Thymus vulgaris]; TvL6H, limonene-6-hydroxylase [Thymus vulgaris]; MgCYP71D18, (-)-(4S)-Limonene-6-hydroxylase [Mentha x gracilis]; MgCYP71D94, Cytochrome P450 71D94 [Mentha x gracilis]; McL6H, limonene-6-hydroxylase [Mentha canadensis]; Mp(-)-L6H, (-)-limonene 6-hydroxylase-like cytochrome p450-dependent oxygenase [Mentha x piperita], MgCYP71D95, 71D95: (-)-(4S)-Limonene-3-hydroxylase [Mentha x gracilis]; MsCYP71D95, 71D95: Limonene-3-hydroxylase [Mentha spicata]; MpCYP71D15, (-)-(4S)-Limonene-3-hydroxylase; AltName: Full = Cytochrome P450 isoform PM2 [Mentha x piperita]; McL3H, (-)P450 limonene-3-hydroxylase [Mentha canadensis]; MaLH, limonene hydroxylase [Mentha arvensis]; Ms(-)-L3H, (-)-limonene 3-hydroxylase-like cytochrome p450-dependent oxygenase [Mentha spicata], MpCYP71D13, Cytochrome P450 71D13; AltName: Full = (-)-(4S)-Limonene-3-hydroxylase; SiCYP71D95-like, P450 71D95-like [Sesamum indicum]; SmCYP71D374; cytochrome P450 [Salvia miltiorrhiza] B) terpene synthesis.The full name of the sequences are TmTPS, gamma-terpinene synthase [Thymus migricus]; TtTPS gamma-terpinene synthase [Thymus trautvetteri]; TpTPS, gamma-terpinene synthase [Thymus pubescens]; TsTPS, putative gamma-terpinene synthase [Thymus serpyllum]; TdTPS, gamma-terpinene synthase [Thymus daenensis]; TvTPS2, terpene synthase 2 [Thymus vulgaris]; TcTPS, gamma-terpinene synthase [Thymus caespititius]; TfTPS, gamma-terpinene synthase [Thymus fedtschenkoi]; TkTPS, gamma-terpinene synthase [Thymus kotschyanus]; OvTPS2, terpene synthase 2 [O. vulgare]; SkTPS2, terpinene synthase [Satureja khuzistanica]; SrTPS2, terpinene synthase, [Satureja rechingeri]; OvTPS5, terpene synthase 5 [O. vulgare]; PvTPS10, terpene synthase 10 [Prunella vulgaris]; SrPS, pinene synthase [Salvia rosmarinus] The ev [file pone.0281351.s003.tif]

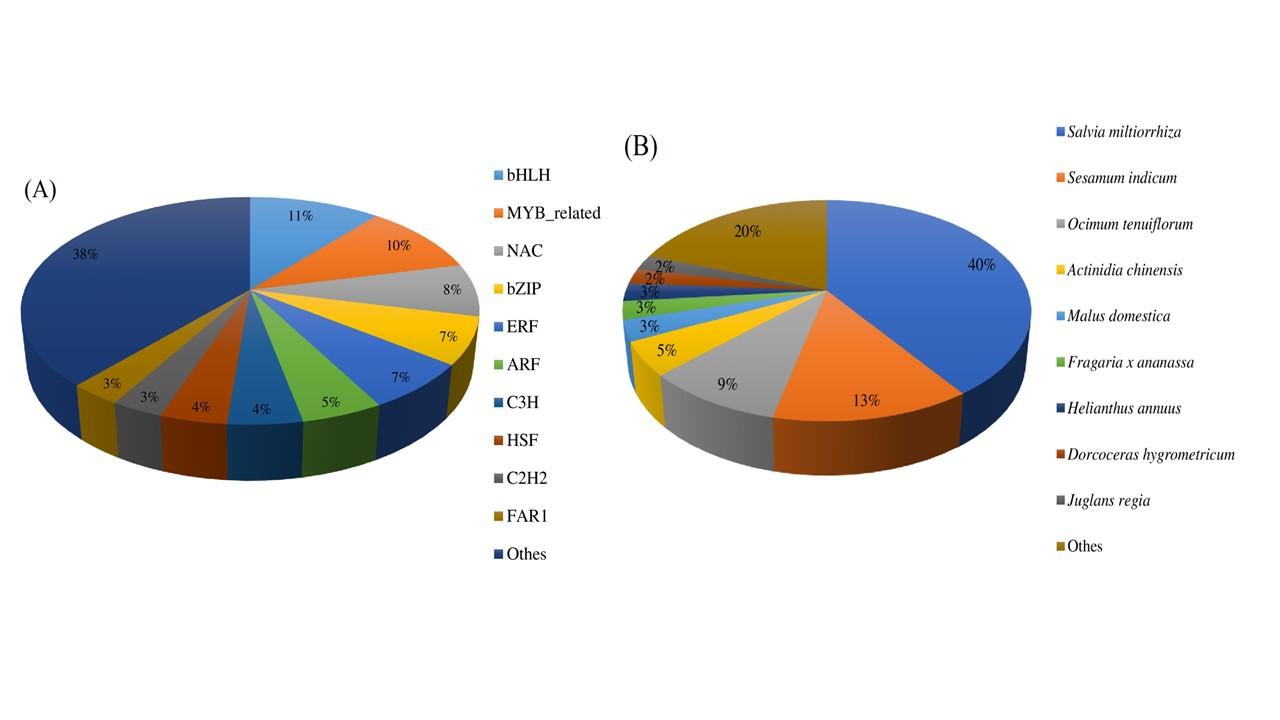

Supplement: S4 Fig — A) Percent of identified TFs families B) Percent of Satureja TFs genes with high homology of plant species. (TIF) [file pone.0281351.s004.tif]

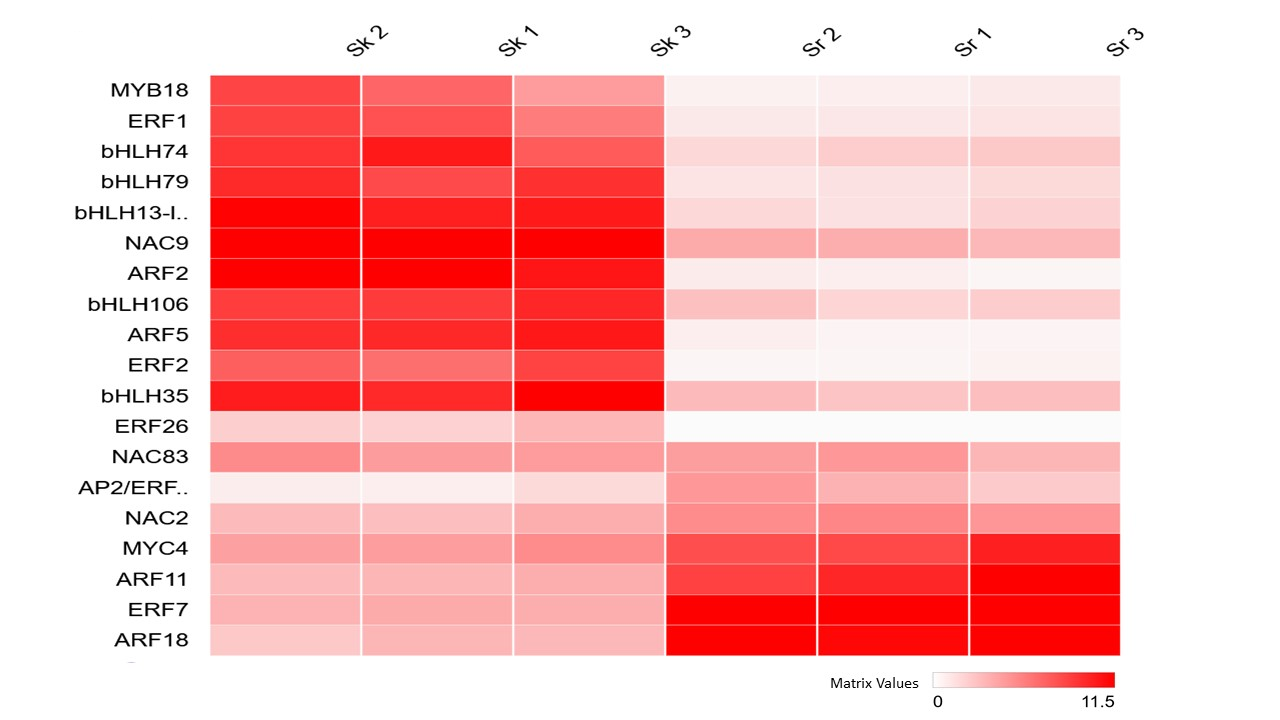

Supplement: S5 Fig — Average of FPKM of all transcripts related to each TF gene was used. Ko numbers show the KEGG maps code related to monoterpene biosynthetic pathway. Specie ID of each replicate is Sk_1: S. khuzistanica_1, Sk_2: S. khuzistanica_2, Sk_3: S. khuzistanica_3, Sr_1: S. rechingeri_1, Sr_2: S. rechingeri _2, Sr_3: S. rechingeri _3. (TIF) [file pone.0281351.s005.tif]
